# Supplementary figures and images for: Phosphorylated vimentin-triggered fibronectin matrix disaggregation enhances the dissemination of Treponema pallidum subsp. pallidum across the microvascular endothelial barrier
Source: PLoS Pathog. 2024 Sep 3;20(9):e1012483. doi: 10.1371/journal.ppat.1012483 (PMC11398692; doi:10.1371/journal.ppat.1012483)

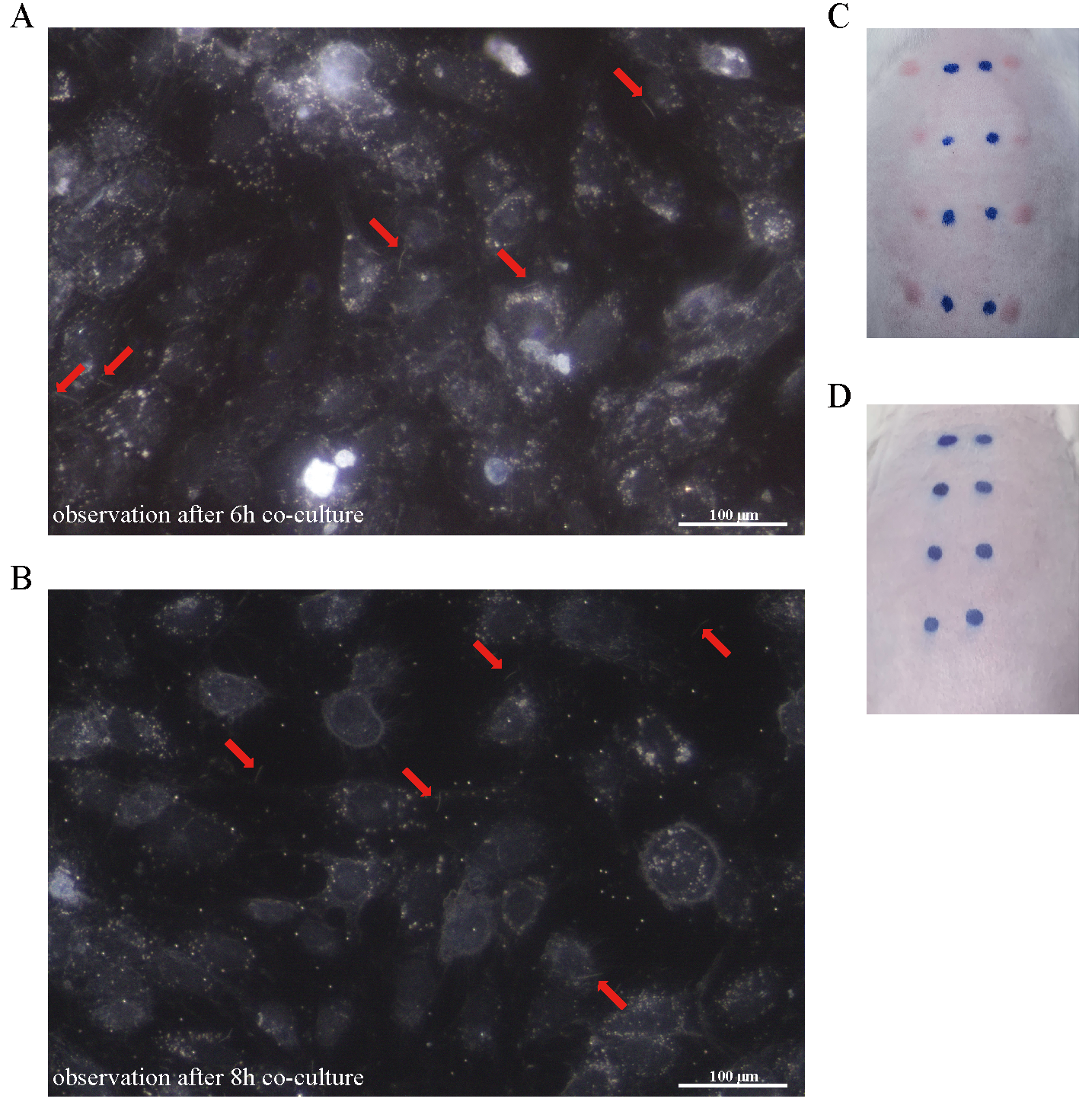

Supplement: S1 Fig — (A-B) Motile Tp after stimulating HMEC-1 cells for (A) 6 hours and (B) 8 hours; observed by DFM under a 400-fold magnification, scale bar = 100 μm. (C) Rabbit back skin, 4 weeks after intradermal injection of live Tp isolated from the 6-hour co-culture medium. (D) Rabbit back skin, 4 weeks after intradermal injection of dead Tp inactivated by the co-cultured medium containing 1% (v/v) penicillin-streptomycin. (TIF) [file ppat.1012483.s001.tif]

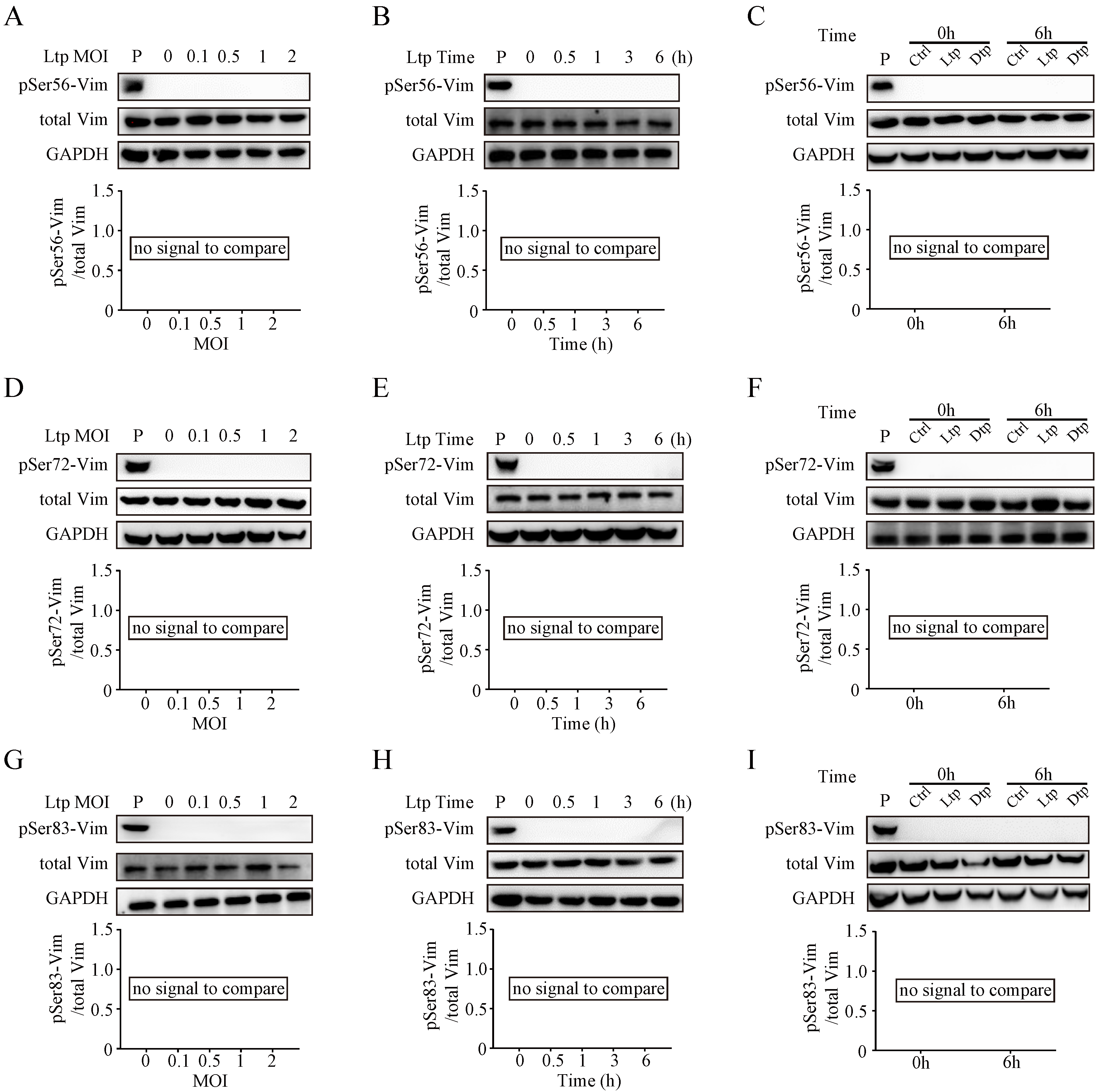

Supplement: S2 Fig — (A-C) Protein expressions of Ser56-phosphorylated (pSer56-) and total vimentin in HMEC-1 (A) after 6 hours of stimulation with live Tp at various MOIs (0, 0.1, 0.5, 1, and 2), (B) after stimulation with live Tp (MOI 2) for different durations (0, 0.5, 1, 3, and 6 hours), and (C) after 0 and 6 hours of stimulation with Ctrl, live Tp (MOI 2), and dead Tp (MOI 2). (D-F) Protein expressions of Ser72-phosphorylated (pSer72-) and total vimentin in HMEC-1 (D) after 6 hours of stimulation with live Tp at various MOIs (0, 0.1, 0.5, 1, and 2), (E) after stimulation with live Tp (MOI 2) for different durations (0, 0.5, 1, 3, and 6 hours), and (F) after 0 and 6 hours of stimulation with Ctrl, live Tp (MOI 2), and dead Tp (MOI 2). (G-I) Protein expressions of Ser83-phosphorylated (pSer83-) and total vimentin in HMEC-1 (G) after 6 hours of stimulation with live Tp at various MOIs (0, 0.1, 0.5, 1, and 2), (H) after stimulation with live Tp (MOI 2) for different durations (0, 0.5, 1, 3, and 6 hours), and (I) after 0 and 6 hours of stimulation with Ctrl, live Tp (MOI 2), and dead Tp (MOI 2). Ctrl: negative control; Ltp: live Tp; Dtp: dead Tp; Vim: vimentin. (TIF) [file ppat.1012483.s002.tif]

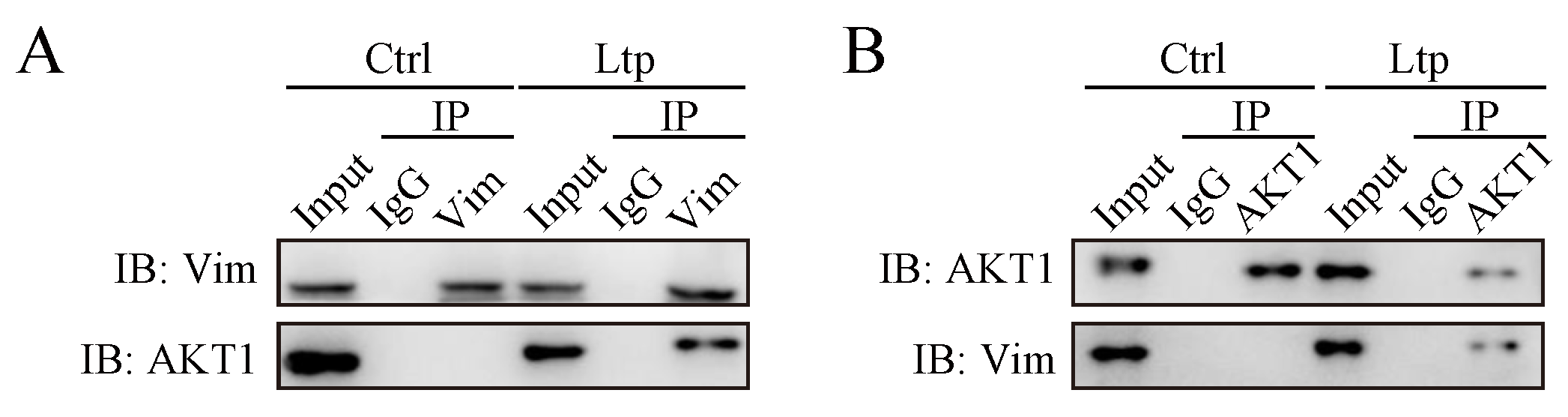

Supplement: S3 Fig — Cell lysates from negative control and live Tp-infected HMEC-1 cells after a 6-hour infection period were subjected to immunoprecipitation using an (A) anti-vimentin antibody or (B) an anti-AKT1 antibody, followed by Western blotting to detect the immunoprecipitated complexes of AKT1 and vimentin. The IgG antibody was used to demonstrate the absence of non-specific binding, and the input lysates (10% of the total lysate) were probed to confirm the presence of both proteins in the lysates. Ctrl: negative control; Ltp: live Tp; IP: immunoprecipitation; IB: immunoblotting; Vim: vimentin. (TIF) [file ppat.1012483.s003.tif]

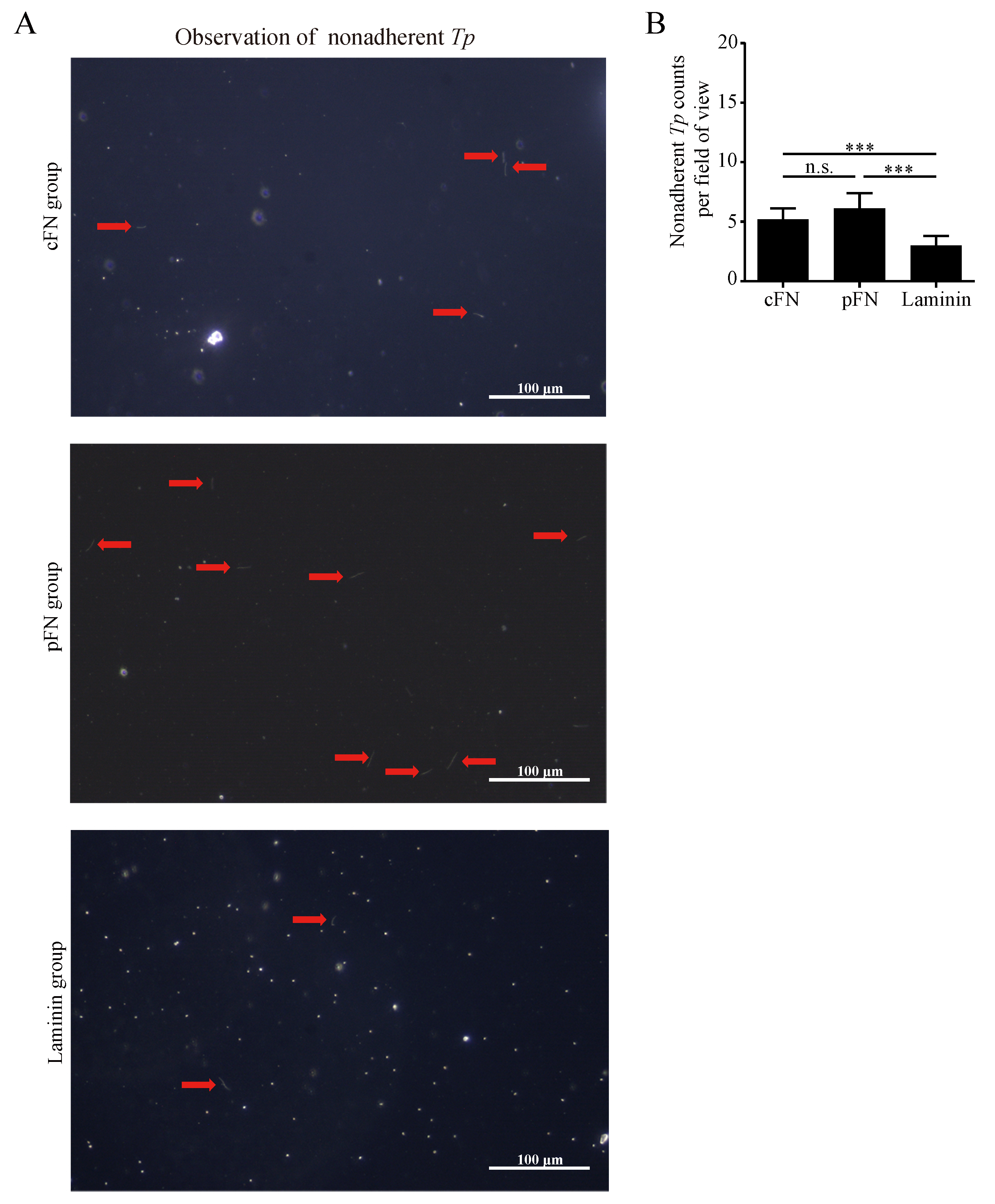

Supplement: S4 Fig — (A) Nonadherent Tp (red arrow) in the supernatant combined with those washed down by PBS, after Tp’s adhesion to cFN, pFN, or laminin for 6 hours; observed by DFM under a 400-fold magnification, scale bar = 100 μm. (B) Quantification of non-adherent Tp per field of view under a 400-fold magnification using DFM. cFN: cellular fibronectin; pFN: plasma fibronectin; n.s.: no significance; ***: p value < 0.001. (TIF) [file ppat.1012483.s004.tif]

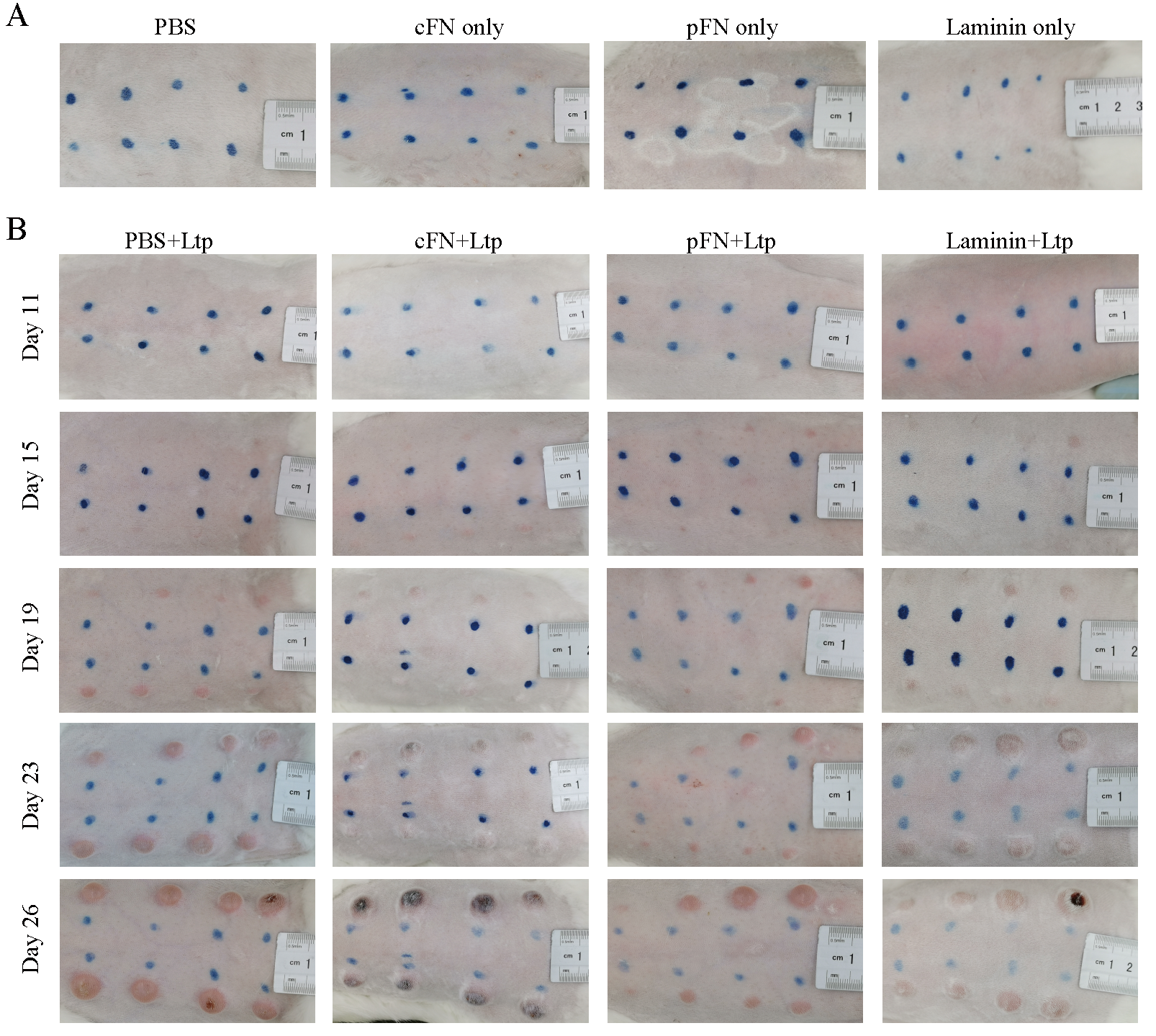

Supplement: S5 Fig — (A) The appearance of rabbit’s skin after the single use of different ECM proteins or PBS, respectively. (B) Changes in Tp-challenged rabbit’s skin at various time points from no evident signs (Day 11) to obvious redness and ulcer (Day 15, 19, and 23) and to sacrifice (Day 26). cFN: cellular fibronectin; pFN: plasma fibronectin; Ltp: live Tp. (TIF) [file ppat.1012483.s005.tif]

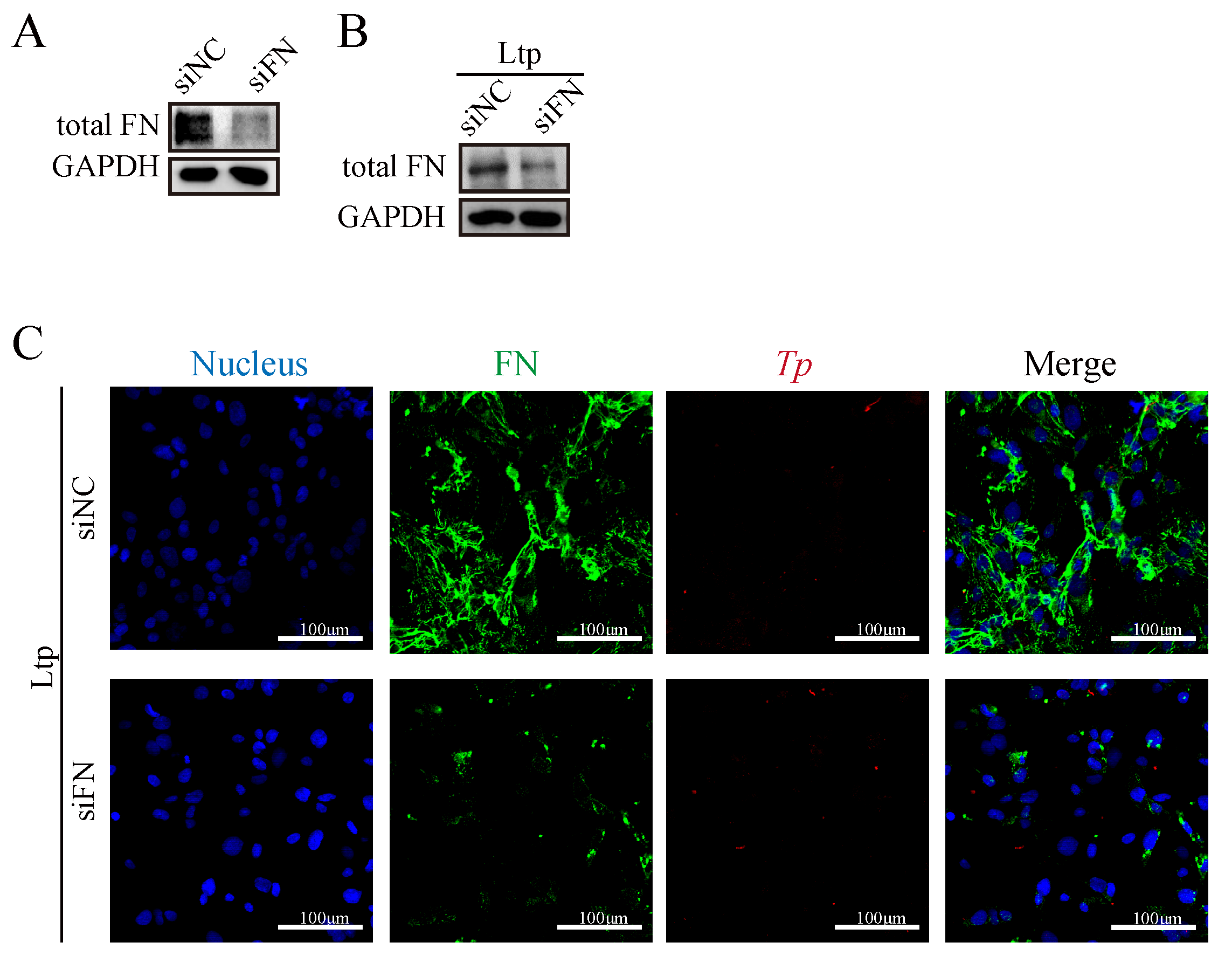

Supplement: S6 Fig — (A) Protein experssions of total fibronectin in HMEC-1 cells with fibronectin knockdown by siRNA (HMEC-1siFN) and cells with scramble siRNA interference as negative control (HMEC-1siNC), respectively. (B) Protein experssions of total fibronectin in HMEC-1 siFN and HMEC-1siNC after stimulation with live Tp (MOI 2) for 6 hours, respectively. (C) Fibronectin matrix of HMEC-1siFN and HMEC-1siNC after stimulation with live Tp (MOI 2) for 6 hours, respectively; observed by fluorescence microscopy, blue for the nucleus, green for the FN matrix, red for Tp, scale bar = 100 μm. NC: negative control; FN: fibronectin; Ltp: live Tp. (TIF) [file ppat.1012483.s006.tif]

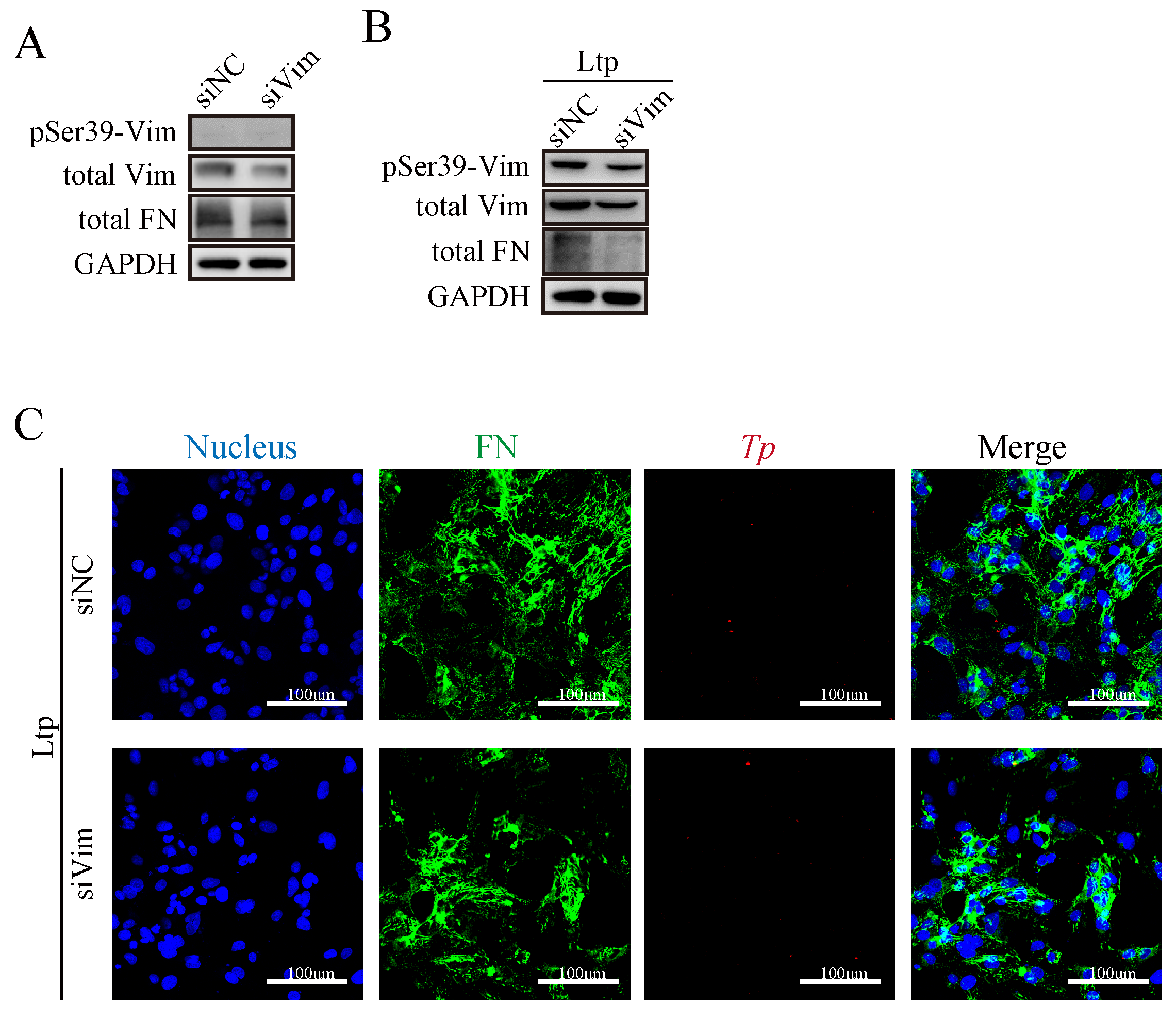

Supplement: S7 Fig — (A) Protein experssions of pSer39-vimentin, total vimentin, and total fibronectin in HMEC-1 cells with vimentin knockdown by siRNA (HMEC-1siVim) and cells with scramble siRNA interference as negative control (HMEC-1siNC), respectively. (B) Protein experssions of pSer39-vimentin, total vimentin, and total fibronectin in HMEC-1siVim and HMEC-1siNC after stimulation with live Tp (MOI 2) for 6 hours, respectively. (C) Fibronectin matrix of HMEC-1siVim and HMEC-1siNC after stimulation with live Tp (MOI 2) for 6 hours, respectively; as observed by fluorescence microscopy; blue for the nucleus, green for the FN matrix, and red for Tp, scale bar = 100 μm. NC: negative control; Vim: vimentin; FN: fibronectin; Ltp: live Tp. (TIF) [file ppat.1012483.s007.tif]

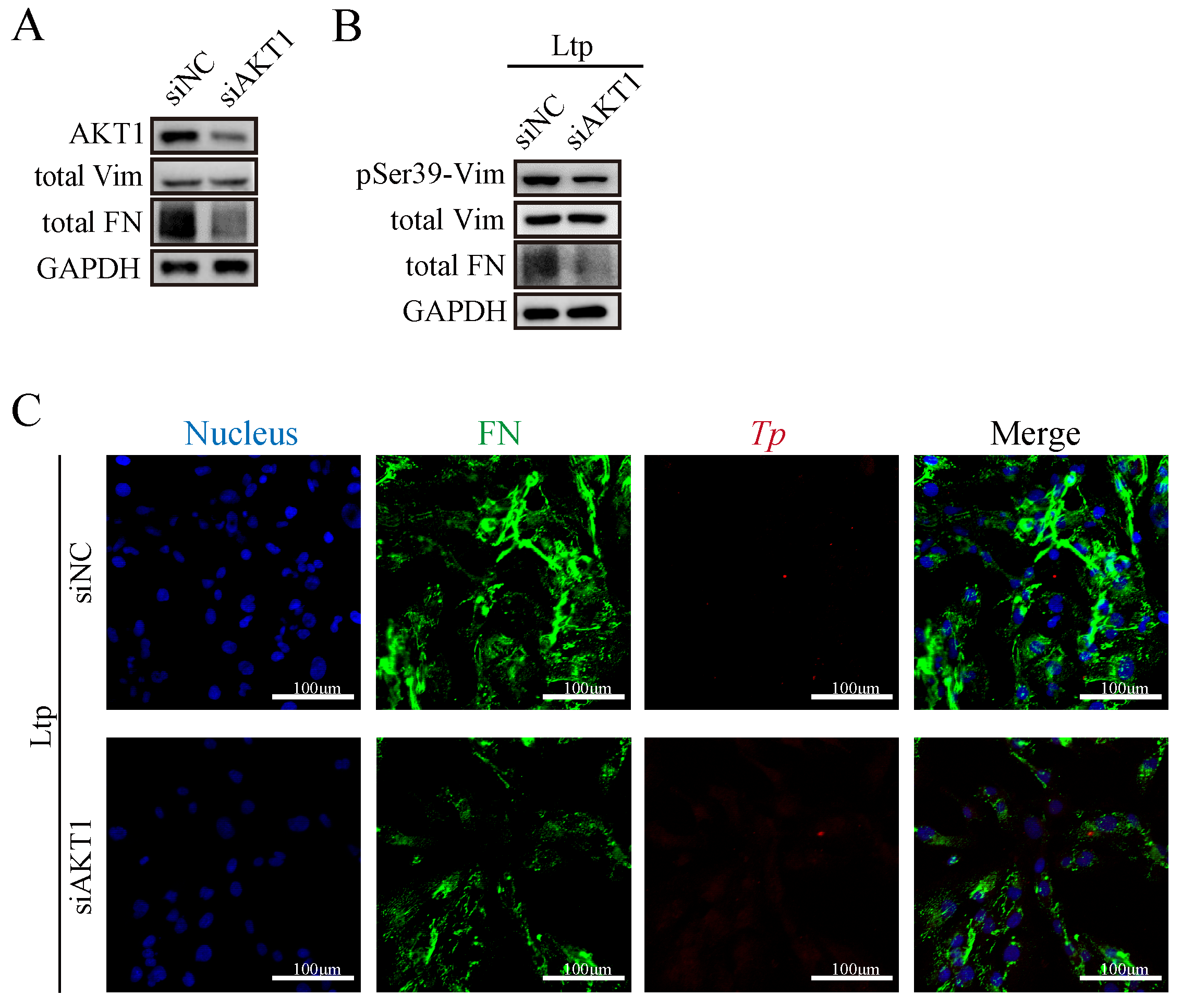

Supplement: S8 Fig — (A) Protein experssions of AKT1, vimentin, and fibronectin in HMEC-1 cells with AKT1 knockdown by siRNA (HMEC-1siAKT1) and cells with scramble siRNA interference as negative control (HMEC-1siNC), respectively. (B) Protein experssions of pSer39-vimentin, total vimentin, and fibronectin in HMEC-1 siAKT1 and HMEC-1siNC after stimulation with live Tp (MOI 2) for 6 hours, respectively. (C) Fibronectin matrix of HMEC-1 siAKT1 and HMEC-1siNC after stimulation with live Tp (MOI 2) for 6 hours, respectively; observed by fluorescence microscopy, blue for the nucleus, green for the FN matrix, red for Tp, scale bar = 100 μm. NC: negative control; Vim: vimentin; FN: fibronectin; Ltp: live Tp. (TIF) [file ppat.1012483.s008.tif]
