# Supplementary material for: Phosphorylated vimentin-triggered fibronectin matrix disaggregation enhances the dissemination of Treponema pallidum subsp. pallidum across the microvascular endothelial barrier
Source: PLoS Pathog. 2024 Sep 3;20(9):e1012483. doi: 10.1371/journal.ppat.1012483 (PMC11398692; doi:10.1371/journal.ppat.1012483)
Supplement: S1 Table — (DOCX) [file ppat.1012483.s009.docx]

**S1 Table. Screening the potential serine/threonine phosphorylation sites on vimentin and their associated protein kinases.**

| **Position** | **Kinase** | **Peptide (phosphorylated site)** | **Score** | **Cutoff** |  |
| --- | --- | --- | --- | --- | --- |
| S39 | AGC/Akt | TTSTRTYSLGSALRP | 0.9962 | 0.8608 |  |
| S39 | AGC/DMPK | TTSTRTYSLGSALRP | 0.9571 | 0.4749 |  |
| S39 | AGC/GRK | TTSTRTYSLGSALRP | 0.0526 | 0.0183 |  |
| S39 | AGC/MAST | TTSTRTYSLGSALRP | 0.0363 | 0.0127 |  |
| S39 | AGC/PKN | TTSTRTYSLGSALRP | 0.7555 | 0.4109 |  |
| S39 | CAMK/CAMK2 | TTSTRTYSLGSALRP | 1 | 0.0117 |  |
| S39 | CAMK/CAMKL | TTSTRTYSLGSALRP | 0.0747 | 0.0098 |  |
| S39 | CAMK/MAPKAPK | TTSTRTYSLGSALRP | 0.9992 | 0.1007 |  |
| S39 | STE/STE-Unique | TTSTRTYSLGSALRP | 0.2859 | 0.1463 |  |
| S39 | TKL/LISK | TTSTRTYSLGSALRP | 0.0371 | 0.029 |  |
| S39 | Atypical/RIO | TTSTRTYSLGSALRP | 0.0441 | 0.0173 |  |
| S39 | Other/Bud32 | TTSTRTYSLGSALRP | 0.0683 | 0.0526 |  |
| S39 | Other/Haspin | TTSTRTYSLGSALRP | 0.0555 | 0.0259 |  |
| S39 | Other/NAK | TTSTRTYSLGSALRP | 0.11 | 0.0997 |  |
| S39 | Other/TLK | TTSTRTYSLGSALRP | 0.1252 | 0.0932 |  |
| S39 | Other/WEE | TTSTRTYSLGSALRP | 0.0243 | 0.0228 |  |
| Serine/threonine kinases were divided into ten groups: AGC, CAMK, CK1, CMGC, PKL, RGC, STE, TKL, Atypical, and Other; and filtrated by their activities on the phosphorylation site of Ser39. | | | | |  |
|  |  |  |  |  |  |
