# Supplementary material for: Phosphorylated vimentin-triggered fibronectin matrix disaggregation enhances the dissemination of Treponema pallidum subsp. pallidum across the microvascular endothelial barrier
Source: PLoS Pathog. 2024 Sep 3;20(9):e1012483. doi: 10.1371/journal.ppat.1012483 (PMC11398692; doi:10.1371/journal.ppat.1012483)
Supplement: S1 File — (A) Alignment by the whole sequences of FN_human and FN_rabbit. (B) Alignment by the individual domains of FN_human and FN_rabbit and the 3D structure prediction. (PDF) [file ppat.1012483.s012.pdf]

S1A File. Alignment by the whole sequences of FN_human and FN_rabbit

```
FN_human  MLRGPGPGLLLLAVQCLGTAVPSTGASKSKRQAQQMVQPQSPVAVSQSKPGCYDNGKHYQINQQWERTYLGNALVCTCYGGSRGFNCESKPEAEETCFDK 100
FN_rabbit --------------------------------------------------GCYDNGKHYQINQQWERTYLGNALVCTCYGGSRGFNCESKPEPEETCFDK 50

FN_human  YTGNTYRVGDTYERPKDSMIWDCTCIGAGRGRISCTIANRCHEGGQSYKIGDTWRRPHETGGYMLECVCLGNGKGEWTCKPIAEKCFDHAAGTSYVVGET 200
FN_rabbit YTGNTYRVGDTYERPKDSMIWDCTCIGAGRGRISCTIANRCHEGGQSYKIGDTWRRPHETGGYMLECVCLGNGKGEWTCKPIAEKCFDHAAGTSYVVGET 150

FN_human  WEKPYQGWMMVDCTCLGEGSGRITCTSRNRCNDQDTRTSYRIGDTWSKKDNRGNLLQCICTGNGRGEWKCERHTSVQTTSSGSGPFTDVRAAVYQPQPHP 300
FN_rabbit WEKPYQGWMMVDCTCLGEGSGRITCTSRNRCNDQDTRTSYRIGDTWSKKDNRGNLLQCICTGNGRGEWKCERHASLQTTSTGSGPFSDVRAAVYQPQPHP 250

FN_human  QPPPYGHCVTDSGVVYSVGMQWLKTQGNKQMLCTCLGNGVSCQETAVTQTYGGNSNGEPCVLPFTYNGRTFYSCTTEGRQDGHLWCSTTSNYEQDQKYSF 400
FN_rabbit QPAPYGHCVTDSGVVYSEGMQWLKTQGNKQMLCTCLGNGVSCQETAVTQTYGGNSNGEPCVLPFTYNGRTFYSCTTEGRQDGHLWCSTTSNYEQDQKYSF 350

FN_human  CTDHTVLVQTRGGNSNGALCHFPFLYNNHNYTDCTSEGRRDNMKWCGTTQNYDADQKFGFCPMAAHEEICTTNEGVMYRIGDQWDKQHDMGHMMRCTCVG 500
FN_rabbit CTDHTVLVQTRGGNSNGALCHFPFLYNNHNYTDCTSEGRRDNMKWCGTTQNYDADQKFGFCPMAAHEEICTTNEGVMYRIGDQWDKQHDMGHMMRCTCVG 450

FN_human  NGRGEWTCIAYSQLRDQCIVDDITYNVNDTFHKRHEEGHMLNCTCFGQGRGRWKCDPVDQCQDSETGTFYQIGDSWEKYVHGVRYQCYCYGRGIGEWHCQ 600
FN_rabbit NGRGEWTCVAYSQLRDQCIVDDITYNVNDTFHKRHEEGHMLNCTCFGQGRGRWKCDPVDQCQDSETRTFYQIGDSWEKYVHGVRYQCYCYGRGIGEWHCQ 550

FN_human  PLQTYPSSSGPVEVFITETPSQPNSHPIQWNAPQPSHISKYILRWRPKNSVGRWKEATIPGHLNSYTIKGLKPGVVYEGQLISIQQYGHQEVTRFDFTTT 700
FN_rabbit PLQTYPGTSGPVQVIITETPSQPNSHPIQWNAPEPSHISKYILRWRPKNSVGRWKEATIPGHLNSYTIKGLRPGVMYEGQLISIQQYGHREVTRFDFTTT 650

FN_human  STSTPVTSNTVTGETTPFSPLVATSESVTEITASSFVVSWVSASDTVSGFRVEYELSEEGDEPQYLDLPSTATSVNIPDLLPGRKYIVNVYQISEDGEQS 800
FN_rabbit STSTPVTSNTVTGETTPLSPVVATSESVTEITASSFVVSWVSASDTVSGFLVEYELSEEGDEPKYLDLPSTVTSVNIPDLLPGRKYIVNVYQISEEGKQS 750

FN_human  LILSTSQTTAPDAPPDTTVDQVDDTSIVVRWSRPQAPITGYRIVYSPSVEGSSTELNLPETANSVTLSDLQPGVQYNITIYAVEENQESTPVVIQQETTG 900
FN_rabbit LILSTSQTTAPDAPPDPTVDQVDDTSIVVRWSRPQAPITGYRVVYSPSVEGSSTELNLPETANSVTLSDLQPGVQYNITIYAVEENQESAPVFIQQETTG 850

FN_human  TPRSDTVPSPRDLQFVEVTDVKVTIMWTPPESAVTGYRVDVIPVNLPGEHGQRLPISRNTFAEVTGLSPGVTYYFKVFAVSHGRESKPLTAQQTTKLDAP 1000
FN_rabbit VPRSDEVPPPKDLQFVEVTDVKVTIMWTPPDSAVTGYRVDVLPVHLPGENGQRLPVSRNTFAEITGLSPGVTYYFKVFAVNHGRESRPLTAQQATKLDAP 950

FN_human  TNLQFVNETDSTVLVRWTPPRAQITGYRLTVGLTRRGQPRQYNVGPSVSKYPLRNLQPASEYTVSLVAIKGNQESPKATGVFTTLQPGSSIPPYNTEVTE 1100
FN_rabbit TNLQFVNETDSSVLVIWTPPRARITGYQLTIGPTRGGQPKQHNVGPTVSKYLLRNLQPGSEYTVTLIAVKGNNQSPKVTGVFTTLQPQSSIPPYSTEVTE 1050

FN_human  TTIVITWTPAPRIGFKLGVRPSQGGEAPREVTSDSGSIVVSGLTPGVEYVYTIQVLRDGQERDAPIVNKVVTPLSPPTNLHLEANPDTGVLTVSWERSTT 1200
FN_rabbit TSIVITWTPAPRIGFKLGVRPSQGGEAPREVTSESGSIVVSGLTPGVEYVYSIQVLRDGKERDAPIVNTVVTPLSPPTNLHLEANPDTGVLTVSWEKSTT 1150

FN_human  PDITGYRITTTPTNGQQGNSLEEVVHADQSSCTFDNLSPGLEYNVSVYTVKDDKESVPISDTIIPEVPQLTDLSFVDITDSSIGLRWTPLNSSTIIGYRI 1300
FN_rabbit PDITGYRITTTPTNGQQGYSLEEVVHADQNSCIFENLSPGLEYNVSVYTVKDDKESVPVSDTIIPEVPQLTDLSFVDITDSSIGLRWTPLNSSTIIGYRI 1250

FN_human  TVVAAGEGIPIFEDFVDSSVGYYTVTGLEPGIDYDISVITLINGGESAPTTLTQQTAVPPPTDLRFTNIGPDTMRVTWAPPPSIDLTNFLVRYSPVKNEE 1400
FN_rabbit TVVAAGEGIPIFEDFVDSSVGYYTVTGLEPGIDYDISVITLINGGESAPTTLTQQTAVPSPTDLRFTNIGPDTMRVTWAPPPSIELTNFLVRYSPVKNEE 1350

FN_human  DVAELSISPSDNAVVLTNLLPGTEYVVSVSSVYEQHESTPLRGRQKTGLDSPTGIDFSDITANSFTVHWIAPRATITGYRIRHHPEHFSGRPREDRVPHS 1500
FN_rabbit DVAELSISPSDNAVVLTNLLPGTEYLVSVSSVYEQHESTPVRGRQKTGLDAPTGIDFSDVTPNSFTVYWTPPRAPITGFWIRHHPEHGVGRPREDRVPPS 1450

FN_human  RNSITLTNLTPGTEYVVSIVALNGREESPLLIGQQSTVSDVPRDLEVVAATPTSLLISWDAPAVTVRYYRITYGETGGNSPVQEFTVPGSKSTATISGLK 1600
FN_rabbit RNSITLTNLNPGTEYVVSIVALNGREQSPPLIGQQSTVSDVPRDLEVIASTPTSLLISWEAPAVTVRYYRITYGETGGNSPVQEFTVPGSKSTATISGLK 1550

FN_human  PGVDYTITVYAVTGRGDSPASSKPISINYRTEIDKPSQMQVTDVQDNSISVKWLPSSSPVTGYRVTTTPKNGPGPTKTKTAGPDQTEMTIEGLQPTVEYV 1700
FN_rabbit PGADYTITVYAVTGRGDSPASSKPISIDYHTEIDKPSQMQVTDVQDNSISVRWLPSSSPVTGYRVTTTPKNGAGPTKTKTAGPDQTEMTIEGLQPTVEYV 1650

FN_human  VSVYAQNPSGESQPLVQTAVTNIDRPKGLAFTDVDVDSIKIAWESPQGQVSRYRVTYSSPEDGIHELFPAPDGEEDTAELQGLRPGSEYTVSVVALHDDM 1800
FN_rabbit VSVYAQNRNGESQPLVQTAVTNIDRPKGLAFTDVDVDSIKIAWESPQGQVSRYRVTYSSPEDGIHELFPAPDGEEDTAELQGLRPGSEYTVSVVALHDDM 1750

FN_human  ESQPLIGTQSTAIPAPTDLKFTQVTPTSLSAQWTPPNVQLTGYRVRVTPKEKTGPMKEINLAPDSSSVVVSGLMVATKYEVSVYALKDTLTSRPAQGVVT 1900
FN_rabbit ESQPLIGTQSTAIPAPTNLKFTQVTPTSLSAQWTPPNVQLTGYRVRVTPKEKTGPMKEINLAPDSSSVVVSGLMVATKYEVSVYALKDTLTSRPVQGVIT 1850

FN_human  TLENVSPPRRARVTDATETTITISWRTKTETITGFQVDAVPANGQTPIQRTIKPDVRSYTITGLQPGTDYKIYLYTLNDNARSSPVVIDASTAIDAPSNL 2000
FN_rabbit TLENVSPPRRARVTDATETTITISWRTKTETITGFRVDAIPANGQNPIQRIIKPDVRSYTITGLQPGTDYKIHLYTLNDNAQSSPVIIDASTAIDAPSNL 1950

FN_human  RFLATTPNSLLVSWQPPRARITGYIIKYEKPGSPPREVVPRPRPGVTEATITGLEPGTEYTIYVIALKNNQKSEPLIGRKKTDELPQLVTLPHPNLHGPE 2100
FN_rabbit HFLATTPNSLLVSWQPPRAKITGYIIKFEKPGSPPREVVPRPRPGVTEATITGLEPGTEYTIYIIALKNNQKSDPLIGRKKTDELPQLVTLPHPNLHGPE 2050

FN_human  ILDVPSTVQKTPFVTHPGYDTGNGIQLPGTSGQQPSVGQQMIFEEHGFRRTTPPTTATPIRHRPRPYPPNVGEEIQIGHIPREDVDYHLYPHGPGLNPNA 2200
FN_rabbit ILDVPSTVQKTPFITNPGYDTGNGIQLPGTSGQQPSVGQQMIFEEHGFRRTTPPTTATPVKLRPRPYLPNVDEDIQIGHVPRGDVDYHLYPHVLGLNPNA 2150

FN_human  STGQEALSQTTISWAPFQDTSEYIISCHPVGTDEEPLQFRVPGTSTSATLTGLTRGATYNVIVEALKDQQRHKVREEVVTVGNSVNEGLNQPTDDSCFDP 2300
FN_rabbit STGQEALSQTTISWTPFQESSEYIISCHPVGTDEQPLQFRVPGTSTSATLTGLTRGATYNIIVEALKDQRRHKVREEVVTVGNSVNEGLNQPTDDSCFDP 2250

FN_human  YTVSHYAVGDEWERMSESGFKLLCQCLGFGSGHFRCDSSRWCHDNGVNYKIGEKWDRQGENGQMMSCTCLGNGKGEFKCDPHEATCYDDGKTYHVGEQWQ 2400
FN_rabbit YTVTHYAVGEEWERLSESGFKLSCQCLGFGSGHFKCDSSKWCHDNGVNYKIGEKWDRQGENGQMMSCTCLGNGKGEFKCDPHEATCYDDGKTYHVGEQWQ 2350

FN_human  KEYLGAICSCTCFGGQRGWRCDNCRRPGGEPSPEGTTGQSYNQYSQRYHQRTNTNVNCPIECFMPLDVQADREDSRE 2477
FN_rabbit KEYLGAICSCTCFGGQRGWRCDNCRRPGVEPSPDSSTGHSYNQYTQRYHQRTNTNVNCPIECFMPLDVQADREDSRE 2427
```

S1B File. Alignment by the individual domains of FN_human and FN_rabbit and the 3D structure prediction

Fibronectin type-I 1

```
sp|P02751|50-90      PGCYDNGKHYQINQQWERTYLGNALVCTCYGGSRGFNCESK  41
sp|Q28749|1-40       -GCYDNGKHYQINQQWERTYLGNALVCTCYGGSRGFNCESK  40
                      ****************************************
```

Fibrin- and heparin-binding 1

```
sp|P02751|52-272     CYDNGKHYQINQQWERTYLGNALVCTCYGGSRGFNCESKPEAEETCFDKYTGNTYRVGDT    60
sp|Q28749|2-222      CYDNGKHYQINQQWERTYLGNALVCTCYGGSRGFNCESKPEPEETCFDKYTGNTYRVGDT    60
                     ***************************************** ******************

sp|P02751|52-272     YERPKDSMIWDCTCIGAGRGRISCTIANRCHEGGQSYKIGDTWRRPHETGGYMLECVCLG    120
sp|Q28749|2-222      YERPKDSMIWDCTCIGAGRGRISCTIANRCHEGGQSYKIGDTWRRPHETGGYMLECVCLG    120
                     ************************************************************

sp|P02751|52-272     NGKGEWTCKPIAEKCFDHAAGTSYVVGETWEKPYQGWMMVDCTCLGEGSGRITCTSRNRC    180
sp|Q28749|2-222      NGKGEWTCKPIAEKCFDHAAGTSYVVGETWEKPYQGWMMVDCTCLGEGSGRITCTSRNRC    180
                     ************************************************************

sp|P02751|52-272     NDQDTRTSYRIGDTWSKKDNRGNLLQCICTGNGRGEWKCER 221
sp|Q28749|2-222      NDQDTRTSYRIGDTWSKKDNRGNLLQCICTGNGRGEWKCER 221
                     *****************************************
```

Fibronectin type-I 2

```
sp|P02751|95-138     ETCFDKYTGNTYRVGDTYERPKDSMIWDCTCIGAGRGRISCTIA    44
sp|Q28749|45-88      ETCFDKYTGNTYRVGDTYERPKDSMIWDCTCIGAGRGRISCTIA    44
                     ********************************************
```

Fibronectin type-I 3

```
sp|P02751|139-182    NRCHEGGQSYKIGDTWRRPHETGGYMLECVCLGNGKGEWTCKPI    44
sp|Q28749|89-132     NRCHEGGQSYKIGDTWRRPHETGGYMLECVCLGNGKGEWTCKPI    44
                     ********************************************
```

Fibronectin type-I 4

```
sp|P02751|184-228    EKCFDHAAGTSYVVGETWEKPYQGWMMVDCTCLGEGSGRITCTSR    45
sp|Q28749|134-178    EKCFDHAAGTSYVVGETWEKPYQGWMMVDCTCLGEGSGRITCTSR    45
                     *********************************************
```

Fibronectin type-I 5

```
sp|P02751|229-273    NRCNDQDTRTSYRIGDTWSKKDNRGNLLQCICTGNGRGEWKCERH    45
sp|Q28749|179-223    NRCNDQDTRTSYRIGDTWSKKDNRGNLLQCICTGNGRGEWKCERH    45
                     *********************************************
```

Fibronectin type-I 6

```
sp|P02751|306-345    GHCVTDSGVVYSVGMQWLKTQGNKQMLCTCLGNGVSCQET 40
sp|Q28749|256-295    GHCVTDSGVVYSEGMQWLKTQGNKQMLCTCLGNGVSCQET 40
                     ************ ***************************
```

Collagen-binding

```
sp|P02751|308-608       CVTDSGVVYSVGMQWLKTQGNKQMLCTCLGNGVSCQETAVTQTYGGNSNGEPCVLPFTYN     60
sp|Q28749|258-558       CVTDSGVVYSEGMQWLKTQGNKQMLCTCLGNGVSCQETAVTQTYGGNSNGEPCVLPFTYN     60
                        ********** *************************************************

sp|P02751|308-608       GRTFYSCTTEGRQDGHLWCSTTSNYEQDQKYSFCTDHTVLVQTRGGNSNGALCHFPFLYN     120
sp|Q28749|258-558       GRTFYSCTTEGRQDGHLWCSTTSNYEQDQKYSFCTDHTVLVQTRGGNSNGALCHFPFLYN     120
                        ************************************************************

sp|P02751|308-608       NHNYTDCTSEGRRDNMKWCGTTQNYDADQKFGFCPMAAHEEICTTNEGVMYRIGDQWDKQ     180
sp|Q28749|258-558       NHNYTDCTSEGRRDNMKWCGTTQNYDADQKFGFCPMAAHEEICTTNEGVMYRIGDQWDKQ     180
                        ************************************************************

sp|P02751|308-608       HDMGHMMRCTCVGNGRGEWTCIAYSQLRDQCIVDDITYNVNDTFHKRHEEGHMLNCTCFG     240
sp|Q28749|258-558       HDMGHMMRCTCVGNGRGEWTCVAYSQLRDQCIVDDITYNVNDTFHKRHEEGHMLNCTCFG     240
                        *********************:**************************************

sp|P02751|308-608       QGRGRWKCDPVDQCQDSETGTFYQIGDSWEKYVHGVRYQCYCYGRGIGEWHCQPLQTYPS     300
sp|Q28749|258-558       QGRGRWKCDPVDQCQDSETRTFYQIGDSWEKYVHGVRYQCYCYGRGIGEWHCQPLQTYPG     300
                        ******************* ***************************************.

sp|P02751|308-608       S        301
sp|Q28749|258-558       T        301
                        :
```

Fibronectin type-II 1

```
sp|P02751|355-403       SNGEPCVLPFTYNGRTFYSCTTEGRQDGHLWCSTTSNYEQDQKYSFCTD       49
sp|Q28749|305-353       SNGEPCVLPFTYNGRTFYSCTTEGRQDGHLWCSTTSNYEQDQKYSFCTD       49
                        *************************************************
```

Fibronectin type-II 2

```
sp|P02751|415-463       SNGALCHFPFLYNNHNYTDCTSEGRRDNMKWCGTTQNYDADQKFGFCPM       49
sp|Q28749|365-413       SNGALCHFPFLYNNHNYTDCTSEGRRDNMKWCGTTQNYDADQKFGFCPM       49
                        *************************************************
```

Critical for collagen binding

```
sp|P02751|464-477       AAHEEICTTNEGVM    14
sp|Q28749|414-427       AAHEEICTTNEGVM    14
                        **************
```

Fibronectin type-I 7

```
sp|P02751|468-511       EICTTNEGVMYRIGDQWDKQHDMGHMMRCTCVGNGRGEWTCIAY      44
sp|Q28749|418-461       EICTTNEGVMYRIGDQWDKQHDMGHMMRCTCVGNGRGEWTCVAY      44
                        *****************************************:**
```

Fibronectin type-I 8

```
sp|P02751|516-558       DQCIVDDITYNVNDTFHKRHEEGHMLNCTCFGQGRGRWKCDPV      43
sp|Q28749|466-508       DQCIVDDITYNVNDTFHKRHEEGHMLNCTCFGQGRGRWKCDPV      43
                        *******************************************
```

Fibronectin type-I 9

```
sp|P02751|559-602       DQCQDSETGTFYQIGDSWEKYVHGVRYQCYCYGRGIGEWHCQPL      44
sp|Q28749|509-552       DQCQDSETRTFYQIGDSWEKYVHGVRYQCYCYGRGIGEWHCQPL      44
                        ******** ***********************************
```

Fibronectin type-III 1

```
sp|P02751|610-702      GPVEVFITETPSQPNSHPIQWNAPQPSHISKYILRWRPKNSVGRWKEATIPGHLNSYTIK    60
sp|Q28749|560-653      GPVQVIITETPSQPNSHPIQWNAPEPSHISKYILRWRPKNSVGRWKEATIPGHLNSYTIK    60
                       ***:*:******************:***********************************

sp|P02751|610-702      GLKPGVVYEGQLISIQQYGHQEVTRFDFTTTST-      93
sp|Q28749|560-653      GLRPGVMYEGQLISIQQYGHREVTRFDFTTTSTS      94
                       **:***:*************:************
```

Fibronectin type-III 2

```
sp|P02751|722-812      -----VATSESVTEITASSFVVSWVSASDTVSGFRVEYELSEEGDEPQYLDLPSTATSVN    55
sp|Q28749|667-762      PLSPVVATSESVTEITASSFVVSWVSASDTVSGFLVEYELSEEGDEPKYLDLPSTVTSVN    60
                            ***************************** ************:*******.****

sp|P02751|722-812      IPDLLPGRKYIVNVYQISEDGEQSLILSTSQTTAPD     91
sp|Q28749|667-762      IPDLLPGRKYIVNVYQISEEGKQSLILSTSQTTAPD     96
                       *******************:*:**************
```

Fibronectin type-III 3

```
sp|P02751|813-904      APPDTTVDQVDDTSIVVRWSRPQAPITGYRIVYSPSVEGSSTELNLPETANSVTLSDLQP    60
sp|Q28749|763-852      APPDPTVDQVDDTSIVVRWSRPQAPITGYRVVYSPSVEGSSTELNLPETANSVTLSDLQP    60
                       **** *************************:*****************************

sp|P02751|813-904      GVQYNITIYAVEENQESTPVVIQQETTGTPRS 92
sp|Q28749|763-852      GVQYNITIYAVEENQESAPVFIQQETTGVP-- 90
                       *****************:**.*******.*
```

Fibronectin type-III 4

```
sp|P02751|909-998      SPRDLQFVEVTDVKVTIMWTPPESAVTGYRVDVIPVNLPGEHGQRLPISRNTFAEVTGLS    60
sp|Q28749|859-948      PPKDLQFVEVTDVKVTIMWTPPDSAVTGYRVDVLPVHLPGENGQRLPVSRNTFAEITGLS    60
                        *:*******************:**********:**:****:*****:*******:****

sp|P02751|909-998      PGVTYYFKVFAVSHGRESKPLTAQQTTKLD    90
sp|Q28749|859-948      PGVTYYFKVFAVNHGRESRPLTAQQATKLD    90
                       ************.*****:******:****
```

Fibronectin type-III 5

```
sp|P02751|999-1088     APTNLQFVNETDSTVLVRWTPPRAQITGYRLTVGLTRRGQPRQYNVGPSVSKYPLRNLQP    60
sp|Q28749|949-1038     APTNLQFVNETDSSVLVIWTPPRARITGYQLTIGPTRGGQPKQHNVGPTVSKYLLRNLQP    60
                       *************:*** ******:****:**:* ** ***:*:****:**** ******

sp|P02751|999-1088     ASEYTVSLVAIKGNQESPKATGVFTTLQPG  90
sp|Q28749|949-1038     GSEYTVTLIAVKGNNQSPKVTGVFTTLQPQ  90
                       .*****:*:*:***::***.*********
```

Fibronectin type-III 6

```
sp|P02751|1089-1175      SSIPPYNTEVTETTIVITWTPAPRIGFKLGVRPSQGGEAPREVTSDSGSIVVSGLTPGVE    60
sp|Q28749|1039-1125      SSIPPYSTEVTETSIVITWTPAPRIGFKLGVRPSQGGEAPREVTSESGSIVVSGLTPGVE    60
                         ******.******:*******************************:**************

sp|P02751|1089-1175      YVYTIQVLRDGQERDAPIVNKVVTPLS    87
sp|Q28749|1039-1125      YVYSIQVLRDGKERDAPIVNTVVTPLS    87
                         ***:*******:********.******
```

Fibronectin type-III 7

```
sp|P02751|1176-1270      PPTNLHLEANPDTGVLTVSWERSTTPDITGYRITTTPTNGQQGNSLEEVVHADQSSCTFD    60
sp|Q28749|1126-1220      PPTNLHLEANPDTGVLTVSWEKSTTPDITGYRITTTPTNGQQGYSLEEVVHADQNSCIFE    60
                         *********************:********************* **********.** *:

sp|P02751|1176-1270      NLSPGLEYNVSVYTVKDDKESVPISDTIIPEVPQL    95
sp|Q28749|1126-1220      NLSPGLEYNVSVYTVKDDKESVPVSDTIIPEVPQL    95
                         ***********************:***********
```

Fibronectin type-III 8; extra domain B

```
sp|P02751|1271-1359      TDLSFVDITDSSIGLRWTPLNSSTIIGYRITVVAAGEGIPIFEDFVDSSVGYYTVTGLEP    60
sp|Q28749|1221-1309      TDLSFVDITDSSIGLRWTPLNSSTIIGYRITVVAAGEGIPIFEDFVDSSVGYYTVTGLEP    60
                         ************************************************************

sp|P02751|1271-1359      GIDYDISVITLINGGESAPTTLTQQTAVP  89
sp|Q28749|1221-1309      GIDYDISVITLINGGESAPTTLTQQTAVP  89
                         *****************************
```

Cell-attachment

```
sp|Q28749|1308-1581      VPSPTDLRFTNIGPDTMRVTWAPPPSIELTNFLVRYSPVKNEEDVAELSISPSDNAVVLT    60
sp|P02751|1358-1631      VPPPTDLRFTNIGPDTMRVTWAPPPSIDLTNFLVRYSPVKNEEDVAELSISPSDNAVVLT    60
                         ** ************************:********************************

sp|Q28749|1308-1581      NLLPGTEYLVSVSSVYEQHESTPVRGRQKTGLDAPTGIDFSDVTPNSFTVYWTPPRAPIT    120
sp|P02751|1358-1631      NLLPGTEYVVSVSSVYEQHESTPLRGRQKTGLDSPTGIDFSDITANSFTVHWIAPRATIT    120
                         ********:**************:*********:********:* *****:*  *** **

sp|Q28749|1308-1581      GFWIRHHPEHGVGRPREDRVPPSRNSITLTNLNPGTEYVVSIVALNGREQSPPLIGQQST    180
sp|P02751|1358-1631      GYRIRHHPEHFSGRPREDRVPHSRNSITLTNLTPGTEYVVSIVALNGREESPLLIGQQST    180
                         *: *******  ********* **********.****************:** *******

sp|Q28749|1308-1581      VSDVPRDLEVIASTPTSLLISWEAPAVTVRYYRITYGETGGNSPVQEFTVPGSKSTATIS    240
sp|P02751|1358-1631      VSDVPRDLEVVAATPTSLLISWDAPAVTVRYYRITYGETGGNSPVQEFTVPGSKSTATIS    240
                         **********:*:*********:*************************************

sp|Q28749|1308-1581      GLKPGADYTITVYAVTGRGDSPASSKPISIDYHT     274
sp|P02751|1358-1631      GLKPGVDYTITVYAVTGRGDSPASSKPISINYRT     274
                         *****.************************:*:*
```

Fibronectin type-III 9

```
sp|P02751|1360-1452      PPTDLRFTNIGPDTMRVTWAPPPSIDLTNFLVRYSPVKNEEDVAELSISPSDNAVVLTNL    60
sp|Q28749|1310-1402      SPTDLRFTNIGPDTMRVTWAPPPSIELTNFLVRYSPVKNEEDVAELSISPSDNAVVLTNL    60
                          ************************:**********************************

sp|P02751|1360-1452      LPGTEYVVSVSSVYEQHESTPLRGRQKTGLDSP      93
sp|Q28749|1310-1402      LPGTEYLVSVSSVYEQHESTPVRGRQKTGLDAP      93
                         ******:**************:*********:*
```

Fibronectin type-III 10

```
sp|P02751|1453-1540      TGIDFSDITANSFTVHWIAPRATITGYRIRHHPEHFSGRPREDRVPHSRNSITLTNLTPG    60
sp|Q28749|1403-1490      TGIDFSDVTPNSFTVYWTPPRAPITGFWIRHHPEHGVGRPREDRVPPSRNSITLTNLNPG    60
                         *******:* *****:*  *** ***: *******  ********* **********.**

sp|P02751|1453-1540      TEYVVSIVALNGREESPLLIGQQSTVSD   88
sp|Q28749|1403-1490      TEYVVSIVALNGREQSPPLIGQQSTVSD   88
                         **************:** **********
```

Fibronectin type-III 11

```
sp|P02751|1541-1634      VPRDLEVVAATPTSLLISWDAPAVTVRYYRITYGETGGNSPVQEFTVPGSKSTATISGLK    60
sp|Q28749|1491-1584      VPRDLEVIASTPTSLLISWEAPAVTVRYYRITYGETGGNSPVQEFTVPGSKSTATISGLK    60
                         *******:*:*********:****************************************

sp|P02751|1541-1634      PGVDYTITVYAVTGRGDSPASSKPISINYRTEID      94
sp|Q28749|1491-1584      PGADYTITVYAVTGRGDSPASSKPISIDYHTEID      94
                         **.************************:*:****
```

Cell attachment site

```
sp|P02751|1615-1617      RGD     3
sp|Q28749|1565-1567      RGD     3
                         ***
```

Fibronectin type-III 12

```
sp|P02751|1635-1726      KPSQMQVTDVQDNSISVKWLPSSSPVTGYRVTTTPKNGPGPTKTKTAGPDQTEMTIEGLQ    60
sp|Q28749|1585-1676      KPSQMQVTDVQDNSISVRWLPSSSPVTGYRVTTTPKNGAGPTKTKTAGPDQTEMTIEGLQ    60
                         *****************:******************** *********************

sp|P02751|1635-1726      PTVEYVVSVYAQNPSGESQPLVQTAVTNIDRP        92
sp|Q28749|1585-1676      PTVEYVVSVYAQNRNGESQPLVQTAVTNIDRP        92
                         ************* .*****************
```

Disordered

```
sp|P02751|1660-1684      VTGYRVTTTPKNGPGPTKTKTAGPD       25
sp|Q28749|1610-1634      VTGYRVTTTPKNGAGPTKTKTAGPD       25
                         ************* ***********
```

Fibronectin type-III 13; extra domain A

```
sp|P02751|1727-1814      KGLAFTDVDVDSIKIAWESPQGQVSRYRVTYSSPEDGIHELFPAPDGEEDTAELQGLRPG   60
sp|Q28749|1677-1764      KGLAFTDVDVDSIKIAWESPQGQVSRYRVTYSSPEDGIHELFPAPDGEEDTAELQGLRPG   60
                         ************************************************************

sp|P02751|1727-1814      SEYTVSVVALHDDMESQPLIGTQSTAIP   88
sp|Q28749|1677-1764      SEYTVSVVALHDDMESQPLIGTQSTAIP   88
                         ****************************
```

Heparin-binding 2

```
sp|P02751|1812-2082      AIPAPTDLKFTQVTPTSLSAQWTPPNVQLTGYRVRVTPKEKTGPMKEINLAPDSSSVVVS   60
sp|Q28749|1762-2032      AIPAPTNLKFTQVTPTSLSAQWTPPNVQLTGYRVRVTPKEKTGPMKEINLAPDSSSVVVS   60
                         ******:*****************************************************

sp|P02751|1812-2082      GLMVATKYEVSVYALKDTLTSRPAQGVVTTLENVSPPRRARVTDATETTITISWRTKTET   120
sp|Q28749|1762-2032      GLMVATKYEVSVYALKDTLTSRPVQGVITTLENVSPPRRARVTDATETTITISWRTKTET   120
                         ***********************.***:********************************

sp|P02751|1812-2082      ITGFQVDAVPANGQTPIQRTIKPDVRSYTITGLQPGTDYKIYLYTLNDNARSSPVVIDAS   180
sp|Q28749|1762-2032      ITGFRVDAIPANGQNPIQRIIKPDVRSYTITGLQPGTDYKIHLYTLNDNAQSSPVIIDAS   180
                         ****:***:*****.**** *********************:********:****:****

sp|P02751|1812-2082      TAIDAPSNLRFLATTPNSLLVSWQPPRARITGYIIKYEKPGSPPREVVPRPRPGVTEATI   240
sp|Q28749|1762-2032      TAIDAPSNLHFLATTPNSLLVSWQPPRAKITGYIIKFEKPGSPPREVVPRPRPGVTEATI   240
                         *********:******************:*******:***********************

sp|P02751|1812-2082      TGLEPGTEYTIYVIALKNNQKSEPLIGRKKT        271
sp|Q28749|1762-2032      TGLEPGTEYTIYIIALKNNQKSDPLIGRKKT        271
                         ************:*********:********
```

Fibronectin type-III 14

```
sp|P02751|1815-1908      APTDLKFTQVTPTSLSAQWTPPNVQLTGYRVRVTPKEKTGPMKEINLAPDSSSVVVSGLM   60
sp|Q28749|1765-1858      APTNLKFTQVTPTSLSAQWTPPNVQLTGYRVRVTPKEKTGPMKEINLAPDSSSVVVSGLM   60
                         ***:********************************************************

sp|P02751|1815-1908      VATKYEVSVYALKDTLTSRPAQGVVTTLENVSPP     94
sp|Q28749|1765-1858      VATKYEVSVYALKDTLTSRPVQGVITTLENVSPP     94
                         ********************.***:*********
```

Binds to FBLN1

```
sp|P02751|1904-2082      NVSPPRRARVTDATETTITISWRTKTETITGFQVDAVPANGQTPIQRTIKPDVRSYTITG   60
sp|Q28749|1854-2032      NVSPPRRARVTDATETTITISWRTKTETITGFRVDAIPANGQNPIQRIIKPDVRSYTITG   60
                         ********************************:***:*****.**** ************

sp|P02751|1904-2082      LQPGTDYKIYLYTLNDNARSSPVVIDASTAIDAPSNLRFLATTPNSLLVSWQPPRARITG   120
sp|Q28749|1854-2032      LQPGTDYKIHLYTLNDNAQSSPVIIDASTAIDAPSNLHFLATTPNSLLVSWQPPRAKITG   120
                         *********:********:****:*************:******************:***

sp|P02751|1904-2082      YIIKYEKPGSPPREVVPRPRPGVTEATITGLEPGTEYTIYVIALKNNQKSEPLIGRKKT   179
sp|Q28749|1854-2032      YIIKFEKPGSPPREVVPRPRPGVTEATITGLEPGTEYTIYIIALKNNQKSDPLIGRKKT   179
                         ****:***********************************:*********:********
```

Fibronectin type-III 15

```
sp|P02751|1909-1995      RRARVTDATETTITISWRTKTETITGFQVDAVPANGQTPIQRTIKPDVRSYTITGLQPGT    60
sp|Q28749|1859-1945      RRARVTDATETTITISWRTKTETITGFRVDAIPANGQNPIQRIIKPDVRSYTITGLQPGT    60
                         ***************************:***:*****.**** *****************

sp|P02751|1909-1995      DYKIYLYTLNDNARSSPVVIDASTAID    87
sp|Q28749|1859-1945      DYKIHLYTLNDNAQSSPVIIDASTAID    87
                         ****:********:****:********
```

Fibronectin type-III 16

```
sp|P02751|1996-2086      APSNLRFLATTPNSLLVSWQPPRARITGYIIKYEKPGSPPREVVPRPRPGVTEATITGLE    60
sp|Q28749|1946-2036      APSNLHFLATTPNSLLVSWQPPRAKITGYIIKFEKPGSPPREVVPRPRPGVTEATITGLE    60
                         *****:******************:*******:***************************

sp|P02751|1996-2086      PGTEYTIYVIALKNNQKSEPLIGRKKTDELP        91
sp|Q28749|1946-2036      PGTEYTIYIIALKNNQKSDPLIGRKKTDELP        91
                         ********:*********:************
```

V region (type III connecting segment, IIICS)

```
sp|P02751|2083-2202      DELPQLVTLPHPNLHGPEILDVPSTVQKTPFVTHPGYDTGNGIQLPGTSGQQPSVGQQMI    60
sp|Q28749|2033-2152      DELPQLVTLPHPNLHGPEILDVPSTVQKTPFITNPGYDTGNGIQLPGTSGQQPSVGQQMI    60
                         *******************************:*:**************************

sp|P02751|2083-2202      FEEHGFRRTTPPTTATPIRHRPRPYPPNVGEEIQIGHIPREDVDYHLYPHGPGLNPNAST    120
sp|Q28749|2033-2152      FEEHGFRRTTPPTTATPVKLRPRPYLPNVDEDIQIGHVPRGDVDYHLYPHVLGLNPNAST    120
                         *****************:: ***** ***.*:*****:** *********  ********
```

Fibronectin type-III 17

```
sp|P02751|2194-2288      PGLNPNASTGQEALSQTTISWAPFQDTSEYIISCHPVGTDEEPLQFRVPGTSTSATLTGL    60
sp|Q28749|2144-2238      LGLNPNASTGQEALSQTTISWTPFQESSEYIISCHPVGTDEQPLQFRVPGTSTSATLTGL    60
                          ********************:***::**************:******************

sp|P02751|2194-2288      TRGATYNVIVEALKDQQRHKVREEVVTVGNSVNEG    95
sp|Q28749|2144-2238      TRGATYNIIVEALKDQRRHKVREEVVTVGNSVNEG    95
                         *******:********:******************
```

Fibronectin type-I 10

```
sp|P02751|2295-2339      DSCFDPYTVSHYAVGDEWERMSESGFKLLCQCLGFGSGHFRCDSS   45
sp|Q28749|2245-2289      DSCFDPYTVTHYAVGEEWERLSESGFKLSCQCLGFGSGHFKCDSS   45
                         *********:*****:****:******* ***********:****
```

Fibrin-binding 2

```
sp|P02751|2297-2428      CFDPYTVSHYAVGDEWERMSESGFKLLCQCLGFGSGHFRCDSSRWCHDNGVNYKIGEKWD    60
sp|Q28749|2247-2378      CFDPYTVTHYAVGEEWERLSESGFKLSCQCLGFGSGHFKCDSSKWCHDNGVNYKIGEKWD    60
                         *******:*****:****:******* ***********:****:****************

sp|P02751|2297-2428      RQGENGQMMSCTCLGNGKGEFKCDPHEATCYDDGKTYHVGEQWQKEYLGAICSCTCFGGQ    120
sp|Q28749|2247-2378      RQGENGQMMSCTCLGNGKGEFKCDPHEATCYDDGKTYHVGEQWQKEYLGAICSCTCFGGQ    120
                         ************************************************************

sp|P02751|2297-2428      RGWRCDNCRRPG   132
sp|Q28749|2247-2378      RGWRCDNCRRPG   132
                         ************
```

Fibronectin type-I 11

```
sp|P02751|2340-2382        RWCHDNGVNYKIGEKWDRQGENGQMMSCTCLGNGKGEFKCDPH    43
sp|Q28749|2290-2332        KWCHDNGVNYKIGEKWDRQGENGQMMSCTCLGNGKGEFKCDPH    43
                           :******************************************
```

Fibronectin type-I 12

```
sp|P02751|2384-2427        ATCYDDGKTYHVGEQWQKEYLGAICSCTCFGGQRGWRCDNCRRP   44
sp|Q28749|2334-2374        ATCYDDGKTYHVGEQWQKEYLGAICSCTCFGGQRGWRCDNC---   41
                           *****************************************
```

P02751: FN_human 2477aa

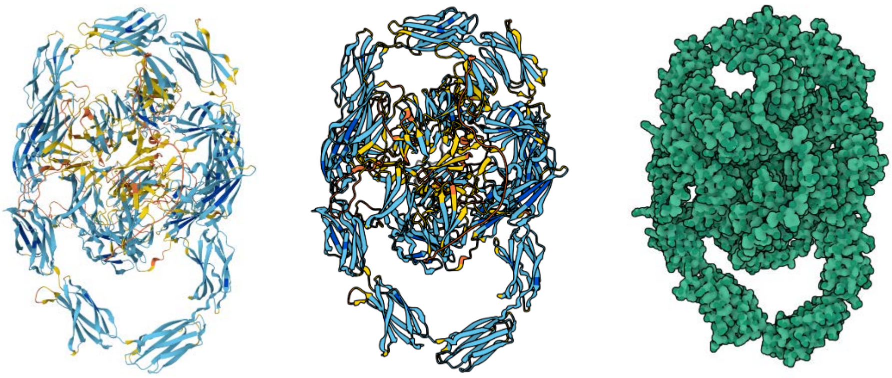

Q28749: FN_rabbit 2427aa (93.8% similar to FN_human)

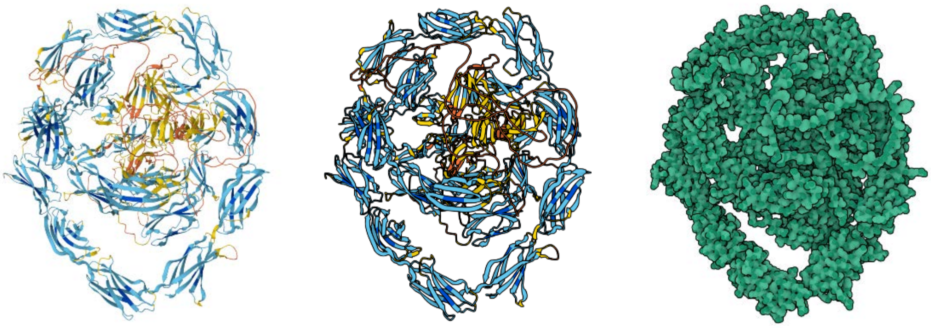

(3D structure by AlphaFold prediction[1,2])

References:

1. Jumper J, Evans R, Pritzel A, Green T, Figurnov M, Ronneberger O, et al. Highly accurate protein structure prediction with AlphaFold. Nature. 2021;596(7873):583-9. doi.org/10.1038/s41586-021-03819-2

2. Varadi M, Anyango S, Deshpande M, Nair S, Natassia C, Yordanova G, et al. AlphaFold Protein Structure Database: massively expanding the structural coverage of protein-sequence space with high-accuracy models. Nucleic acids research. 2021;50(D1):D439-D44. doi.org/10.1093/nar/gkab1061
