# Supplementary material for: Phosphorylated vimentin-triggered fibronectin matrix disaggregation enhances the dissemination of Treponema pallidum subsp. pallidum across the microvascular endothelial barrier
Source: PLoS Pathog. 2024 Sep 3;20(9):e1012483. doi: 10.1371/journal.ppat.1012483 (PMC11398692; doi:10.1371/journal.ppat.1012483)
Supplement: S2 File — (PDF) [file ppat.1012483.s013.pdf]

Methods and materials for gene knockdown experiments by siRNA

HMEC-1 cell culture and gene knockdown by small interfering RNA (siRNA)

Wild-type and siRNA-transfected HMEC-1 cells were cultivated in completed endothelial cell medium (1001; ScienCell, USA) in a humidified atmosphere at 37℃ and 5% CO_2. When the density of HMEC-1 cells seeded in 6-well culture plates reached 80%, cells were replaced with 500.0 μL Opti-MEM medium (31985070; Gibco, USA). Besides, 5.0 μL siRNA (20.0 μM) was added into a tube containing 250.0 μL Opti-MEM medium, and 5.0 μL Lipofectamine RNAiMAX (13778150; Thermo Fisher, USA) was added into another tube containing 250.0 μL Opti-MEM medium. The contents of the two tubes were then mixed and incubated at room temperature for 20 minutes, following by adding this mixturr dropwise to the cell medium. After 8 hours, the medium was replaced with 2.0 mL fresh completed endothelial cell medium. Follow-up experiments, such as live *Tp* infection, immunofluorescence assay, and western blotting, were performed after an additional 16 hours.

The sequences of siRNA targetted Vimentin (siVim): sense (5'→3') CUGAGUACCGGAGACAGGUdTdT; antisense (5'→3') ACCUGUCUCCGGUACUCAGdTdT. The sequences of siRNA targetted fibronectin (siFN): sense (5'→3') GCAGCACAACUUCGAAUUAdTdT; antisense (5'→3') UAAUUCGAAGUUGUGCUGCdTdT. The sequences of siRNA targetted AKT1 (siAKT1): sense (5'→3') GAACAAUCCGAUUCACGUAdTdT; antisense (5'→3') UACGUGAAUCGGAUUGUUCdTdT. The sequences of scramble siRNA as negative control (siNC): sense (5'→3') UUCUCCGAACGUGUCACGUdTdT; antisense

(5’→3’) ACGUGACACGUUCGGAGAAdTdT.
