# Supplementary material for: Phosphorylated vimentin-triggered fibronectin matrix disaggregation enhances the dissemination of Treponema pallidum subsp. pallidum across the microvascular endothelial barrier
Source: PLoS Pathog. 2024 Sep 3;20(9):e1012483. doi: 10.1371/journal.ppat.1012483 (PMC11398692; doi:10.1371/journal.ppat.1012483)
Supplement: Attachment — Submitted filename: Comments.docx [file ppat.1012483.s015.docx]

In the present study, the authors reported that vimentin may disrupt cFN-integrin binding after Tp infection, facilitating the dissemination of Tp across the MEB. phosphorylated Ser39 of vimentin triggered fibronectin matrix disaggregation and the related mechanisms and domestrated that the stability of the cFN matrix alleviated the dissemination of Tp across the MEB. However, the manuscript need more revision before publication. My comments are as follow:

Major Comments:

1. In this study, to investigate the effect of Tp on microvascular endothelial cells, HMEC-1 cells were co-culture with Tp in vitro to simulate the infection environment of syphilis. However, to our knowledge, the technology of Tp in vitro culture is not yet mature. How can you ensure that Tp can play a role similar to in vivo infection during co-culture with HMEC-1 cells. In addition, although Tp can survive in vitro for a short time, whether it has corresponding infective activity remains unproven.
2. Different MOL of Tp were used for this study, but the specific quantitative method of Tp did not shown to be mentioned in the material method.
3. Result 4. There was no significant difference in PAKT1 level after Tp stimulation with MOL 1 and 2 in Fig4B. Why should the stimulation concentration with Mol 2 be used in subsequent experiments.
4. The Figure of Wb in Fig4F is not clear, please replace it with a clearer Figure.
5. Please detect the stability of cFN matrix and the spread of Tp infection after inhibition the phosphorylation of pSer39.
6. Result 6. Is direct injection of cFN via intradermal injection similar to vaccination? How to ensure the functional structure of the protein in vivo after injected directly without any adjuvants? In addition, The PCR results in different tissues showed that the load of Tp in the PBS+Ltp group was also not high, Whether this indicates that Tp does not diffuse effectively in vivo, which will directly affect the reliability of this result.

Minor Comments:

1. In the section of introduction, the description of research progresss in Fibronectin, extracellular matrix, and microvascular endothelial barrier is too simple and lacks logic, which makes it difficult to agree with the necessity of this study.
2. Line 213, ml should be written in the same way as before.
3. Lin133, and 200, please replace “in vivo” with “*in vivo*”.
4. The red fluorescence of Tp in Fig1A is not obvious.
5. Fig3C: Similarly, Less and not obvious Tp red fluorescence in this figure will affect the reliability of the experiment
