# Supplementary material for: Phosphorylated vimentin-triggered fibronectin matrix disaggregation enhances the dissemination of Treponema pallidum subsp. pallidum across the microvascular endothelial barrier
Source: PLoS Pathog. 2024 Sep 3;20(9):e1012483. doi: 10.1371/journal.ppat.1012483 (PMC11398692; doi:10.1371/journal.ppat.1012483)
Supplement: Attachment — Submitted filename: submit_Response letter -plain text.docx [file ppat.1012483.s016.docx]

Dear Prof. Yang,

Thank you very much for submitting your manuscript "Phosphorylated vimentin-triggered fibronectin matrix disaggregation enhances the dissemination of Treponema pallidum subsp. pallidum across the microvascular endothelial barrier" for consideration at PLOS Pathogens. As with all papers reviewed by the journal, your manuscript was reviewed by members of the editorial board and by several independent reviewers. In light of the reviews (below this email), we would like to invite the resubmission of a significantly-revised version that takes into account the reviewers' comments.

Thank you for submitting your manuscript to PLOS Pathogens. Your study is intriguing and contributes novel insights to the field. After careful consideration, I recommend that the manuscript undergo major revisions.

In addressing the concerns raised, it is crucial to clearly differentiate between the mechanisms of cFN blockade and cFN adhesion affecting Tp's trans-endothelial migration. Your response should include detailed mechanistic explanations or experimental data to support the hypothesis that Tp penetration is inhibited by cFN blockade rather than adhesive interactions. Please consider conducting additional experiments or providing theoretical rationale backed by literature.

Another critical issue is the absence of co-immunoprecipitation experiments for AKT and vimentin. The co-localization and potential interaction between these proteins may provide significant insights into the signaling pathways involved in your study's context. I recommend including these experiments to strengthen the manuscript and provide a more comprehensive understanding of the molecular mechanisms at play.

Please address these points thoroughly in your revised manuscript and provide a detailed point-by-point response to each of the reviewer’s comments. We look forward to receiving your revised submission.

We cannot make any decision about publication until we have seen the revised manuscript and your response to the reviewers' comments. Your revised manuscript is also likely to be sent to reviewers for further evaluation.

[1] A letter containing a detailed list of your responses to the review comments and a description of the changes you have made in the manuscript. Please note while forming your response, if your article is accepted, you may have the opportunity to make the peer review history publicly available. The record will include editor decision letters (with reviews) and your responses to reviewer comments. If eligible, we will contact you to opt in or out.

[2] Two versions of the revised manuscript: one with either highlights or tracked changes denoting where the text has been changed; the other a clean version (uploaded as the manuscript file).

Important additional instructions are given below your reviewer comments.

Please prepare and submit your revised manuscript within 60 days. If you anticipate any delay, please let us know the expected resubmission date by replying to this email. Please note that revised manuscripts received after the 60-day due date may require evaluation and peer review similar to newly submitted manuscripts.

Thank you again for your submission. We hope that our editorial process has been constructive so far, and we welcome your feedback at any time. Please don't hesitate to contact us if you have any questions or comments.

Sincerely,

yimou wu

Guest Editor

PLOS Pathogens

Matthew Wolfgang

Section Editor

PLOS Pathogens

Michael Malim

Editor-in-Chief
PLOS Pathogens

orcid.org/0000-0002-7699-2064

RESPONSE:

Dear Prof. Malim, Prof. Wolfgang, and Prof. Wu,

Thank you very much for the opportunity to revise and resubmit our manuscript (number: PPATHOGENS-D-24-00489) titled “Phosphorylated vimentin-triggered fibronectin matrix disaggregation enhances the dissemination of Treponema pallidum subsp. pallidum across the microvascular endothelial barrier” for consideration at PLOS Pathogens.

We deeply appreciate the insightful and constructive feedback provided by you and reviewers. We acknowledge the significance of the concerns raised and are committed to addressing all the points thoroughly via providing more detailed mechanistic explanations and additional experimental data to strengthen our manuscript.

We are grateful for this opportunity and value the feedback and guidance provided. Below, we have provided a detailed point-by-point response (in blue font) to each of the reviewers' comments, along with the corresponding changes (both in figures and text) made to a significantly revised version of the manuscript (with highlights in blue font). We believe that these revisions will substantially enhance the quality and comprehensiveness of our study.

Best regards,

Bin Yang

Dermatology Hospital, Southern Medical University, Guangzhou, China

Email: yangbin1@smu.edu.cn
***********************

Thank you for submitting your manuscript to PLOS Pathogens. Your study is intriguing and contributes novel insights to the field. After careful consideration, I recommend that the manuscript undergo major revisions.

In addressing the concerns raised, it is crucial to clearly differentiate between the mechanisms of cFN blockade and cFN adhesion affecting Tp's trans-endothelial migration. Your response should include detailed mechanistic explanations or experimental data to support the hypothesis that Tp penetration is inhibited by cFN blockade rather than adhesive interactions. Please consider conducting additional experiments or providing theoretical rationale backed by literature.

RESPONSE:

Thank you for raising this important issue. We have conducted an experiment aimed at addressing the concern that the impediment to *Tp* penetration may be primarily attributed to the dense structure of the cFN matrix rather than its adhesive properties. Detailed explanations have been included in the Result section and Discussion section of revised manuscript.

(a) In both the *in vitro* endothelial barrier traversal experiment and *in vivo* dissemination experiment, the outcomes of the cFN+Ltp group (compared with those of the pFN+Ltp group or the Laminin+Ltp group) revealed a rescue of the FN matrix disaggregation due to cFN pretreatment, indicating that the dense FN matrix significantly hindered *Tp* from crossing the endothelial barrier and disseminating (Fig 5 & Fig 6). Previous studies^[1-3]^ have documented *Tp*'s adhesive effects on cFN, pFN, and laminin; however, our findings demonstrate that *Tp* exhibits maximal adherence to laminin instead of cFN (S4 Fig). By integrating these results with existing data, we established that while *Tp* can adhere to both laminin and FN, there exists a notable disparity in its ability to traverse the endothelial barrier. Preliminarily, we speculate that *Tp* 's adherence to ECM proteins may not directly correlate with the disaggregation of the cFN matrix.

(b) We acknowledge that *Tp* may utilize its surface proteins to bind fibronectin and other ECM proteins, thereby promoting vascular interactions, as described in the previous studies^[4,5]^. Due to the challenges associated with gene-editing for *Tp*, these investigations typically employ heterologous expression of *Tp* surface proteins in *Borrelia burgdorferi* to explore the functions of *Tp* proteins. However, this kind of intervention may not fully reflect the natural infection dynamics of *Tp* in our experiments: i) Whether these proteins exhibit the same adhesion properties under conditions of heterologous expression and natural infection remains a topic for further discussion, and no recent studies have demonstrated that overexpression or inhibition of *Tp* proteins can induce structural alterations in the FN matrix surrounding host cells. ii) The adhesion of *Tp* to specific ECM proteins results from the collective action of numerous surface proteins. We believe that fibronectin matrix disaggregation-mediated and fibronectin adhesion-mediated *Tp* dissemination are actually two independent pathways in accelerating the infection process.

Thank you once again for your valuable feedback.

**S4 Fig.** **The adhesion capability of *Tp* to different ECM proteins.** **(A)** Nonadherent Tp (red arrow) in the supernatant combined with those washed down by PBS, after *Tp*’s adhesion to cFN, pFN, or laminin for 6 hours; observed by DFM under a 400-fold magnification, scale bar = 100 μm. **(B)** Quantification of non-adherent *Tp* per field of view under a 400-fold magnification using DFM. cFN: cellular fibronectin; pFN: plasma fibronectin; n.s.: no significance; ***: p value < 0.001.

References of this response:

1.	Fitzgerald TJ, Repesh LA, Blanco DR, Miller JN. Attachment of Treponema pallidum to fibronectin, laminin, collagen IV, and collagen I, and blockage of attachment by immune rabbit IgG. The British journal of venereal diseases. 1984;60(6):357-63. doi.org/10.1136/sti.60.6.357 PMID:6394096.

2.	Fitzgerald TJ, Repesh LA. Interactions of fibronectin with Treponema pallidum. Genitourinary medicine. 1985;61(3):147-55. doi.org/10.1136/sti.61.3.147 PMID:3891582.

3.	Lee JH, Choi HJ, Jung J, Lee MG, Lee JB, Lee KH. Receptors for Treponema pallidum attachment to the surface and matrix proteins of cultured human dermal microvascular endothelial cells. Yonsei medical journal. 2003;44(3):371-8. doi.org/10.3349/ymj.2003.44.3.371 PMID:12833573.

4.	Kao WA, Pětrošová H, Ebady R, Lithgow KV, Rojas P, Zhang Y, et al. Identification of Tp0751 (Pallilysin) as a Treponema pallidum Vascular Adhesin by Heterologous Expression in the Lyme disease Spirochete. Sci Rep. 2017;7(1):1538. doi.org/10.1038/s41598-017-01589-4 PMID:28484210.

5.	Parker ML, Houston S, Pětrošová H, Lithgow KV, Hof R, Wetherell C, et al. The Structure of Treponema pallidum Tp0751 (Pallilysin) Reveals a Non-canonical Lipocalin Fold That Mediates Adhesion to Extracellular Matrix Components and Interactions with Host Cells. PLoS pathogens. 2016;12(9):e1005919. doi.org/10.1371/journal.ppat.1005919 PMID:27683203.

Another critical issue is the absence of co-immunoprecipitation experiments for AKT and vimentin. The co-localization and potential interaction between these proteins may provide significant insights into the signaling pathways involved in your study's context. I recommend including these experiments to strengthen the manuscript and provide a more comprehensive understanding of the molecular mechanisms at play.

RESPONSE:

We appreciate your thoughtful inquiry regarding the inclusion of co-immunoprecipitation (co-IP) experiments for AKT1 and vimentin. We agree that demonstrating the protein-protein interaction between these molecules is crucial for understanding the pAKT1/pSer39-vimentin/fibronectin pathway.

Here, we have successfully demonstrated the interaction between vimentin and AKT1 using co-IP experiments. In our study, vimentin was immunoprecipitated from cell lysates using a specific anti-vimentin antibody. Subsequent Western blotting analysis confirmed the presence of AKT1 in the immunoprecipitated complexes following live *Tp* infection (S3A Fig). Similarly, immunoprecipitation of AKT1 using an anti-AKT1 antibody revealed vimentin in the immunoprecipitated complexes after live *Tp* infection (S3B Fig). These results exhibit a physical interaction between vimentin and AKT1, providing a solid foundation for understanding the specific phosphorylation of vimentin at Ser39 by AKT1. They also validate the reliability of our approach to reduce Ser39 phosphorylation levels using an AKT inhibitor, thereby reversing the downstream effects on Fibronectin matrix disaggregation.

**S3 Fig. Co-immunoprecipitation of vimentin and AKT1.** Cell lysates from negative control and live *Tp*-infected HMEC-1 cells after a 6-hour infection period were subjected to immunoprecipitation using an anti-vimentin antibody **(A)** or an anti-AKT1 antibody **(B)**, followed by Western blotting to detect the immunoprecipitated complexes of AKT1 and vimentin. The IgG antibody was used to demonstrate the absence of non-specific binding, and the input lysates (10% of the total lysate) were probed to confirm the presence of both proteins in the lysates. Ctrl: negative control; Ltp: live *Tp*; IP: immunoprecipitation; IB: immunoblotting; Vim: vimentin.

Please address these points thoroughly in your revised manuscript and provide a detailed point-by-point response to each of the reviewer’s comments. We look forward to receiving your revised submission.


Reviewer's Responses to Questions

**Part I - Summary**

Please use this section to discuss strengths/weaknesses of study, novelty/significance, general execution and scholarship.

Reviewer #1: In the present study, the authors reported that vimentin may disrupt cFN-integrin binding after Tp infection, facilitating the dissemination of Tp across the MEB. phosphorylated Ser39 of vimentin triggered fibronectin matrix disaggregation and the related mechanisms and demonstrated that the stability of the cFN matrix alleviated the dissemination of Tp across the MEB. However, the manuscript needs more revision before publication.

RESPONSE:

Dear Reviewer #1,

Thank you very much for your insightful comments and constructive feedback on our manuscript. We sincerely appreciate your thorough review of our study.

As outlined in our manuscript, we consider the findings from our study to be significant in elucidating the mechanisms involved in *Tp* dissemination across the microvascular endothelial barrier. Your feedback is valuable to us, and we are dedicated to addressing each of your suggestions meticulously to enhance the depth and clarity of our manuscript. We are committed to improving the quality of our study to ensure its scientific rigor and significance.

Best regards

Reviewer #2: The authors present a compelling case that one effect of Treponema pallidum (Tp) infection involves AKT-1 activation and its effects on vimentin filament organization leading to disruption of vascular extracellular matrix (ECM). Vimentin is known to be a stress protein (e.g. see Pattabiraman et al, 2020. Vimentin protects differentiating stem cells from stress. Scientific Reports, 10(1), p.19525) so a range of changes in cellular inputs are likely to be influenced by the state of the vimentin filament network.

The observations seem compelling and medically relevant.

RESPONSE:

Dear Reviewer #2,

Thank you for your positive assessment of our work. Your recognition of the potential involvement of AKT1 activation and its effects on vimentin filament organization in *Tp* infection underscores the significance of our study.

Additionally, we appreciate your reference to the role of vimentin as a stress protein and the likelihood of various cellular inputs being influenced by the state of the vimentin filament network. We believe that understanding these intricate molecular mechanisms can provide valuable insights into the pathogenesis of *Tp* infection and potentially lead to the development of novel therapeutic strategies.

Once again, thank you for your encouraging feedback and for recognizing the importance of our research. We hope that after further improving the manuscript, we can provide new perspectives on how *Tp* penetrates the endothelial barrier, attracting more researchers' attention and promoting extensive studies on this aspect of the mechanism.

Best regards

Reviewer #3: In this manuscript, the authors demonstrate through in vivo and in vitro experiments that live Tp activates pAKT1/pSer39 vimentin signal to expedite the disaggregation of the FN matrix and provide a new perspective on the mechanism by which Tp disrupts the ECM. This manuscript is logically clear, with fluent and understandable language and a detailed methodological presentation. Some details need attention, and it is ready for acceptance after minor modifications.

RESPONSE:

Dear Reviewer #3,

Thank you for your positive feedback and for highlighting the strengths of our manuscript. By elucidating the molecular basis of how *Tp* disrupts the extracellular matrix and interacts with microvascular endothelial cells, we can better understand the pathogenesis of syphilis. This understanding may inform the development of new therapeutic strategies aimed at preventing or mitigating tissue damage caused by this tricky bacterium.

We have carefully considered your suggestions for minor modifications and have made the necessary adjustments to improve the manuscript. Specifically, we have refined our explanations and included additional experimental data where needed to strengthen our conclusions. We are committed to making any further improvements as necessary and greatly appreciate your constructive comments. We hope that the revised manuscript meets your expectations.

Best regards

Reviewer #4: In this study, Luo et al. described a novel molecular basis of T. pallidum to penetrate endothelial cells and aimed to correlate this mechanism to T. pallidum dissemination. While the concept is interesting, some experimental design allows only get the correlation of phenotypes rather than building a cause and results relationship of these phenotypes. Specifically, the current experimental design in the in vivo work would introduce many confounding factors, leading to the results unsupportive to the conclusion made by the authors. Additionally, the writing and clarity may need to be significantly improved by providing more details of how the experiments are performed. My suggestions are as below:

RESPONSE:

Dear Reviewer #4,

Thank you very much for your careful review and helpful remarks that highlight the areas where our manuscript can be improved.

We appreciate your point regarding the correlation of phenotypes and the potential confounding factors in our experimental design. Based on your suggestions, we have conducted additional experiments to establish a more robust cause-and-effect relationship between *T. pallidum* (*Tp*)'s molecular mechanisms and its dissemination. Furthermore, we have acknowledged the need to improve the writing and clarity of our manuscript. We have included more detailed descriptions of our experimental procedures to ensure that the methodology is transparent and easy to follow, and we also have conducted a more detailed interpretation of our *in vivo* experiments. By analyzing various parameters more thoroughly, we aim to provide a clearer and more nuanced understanding of the mechanisms by which *Tp* penetrates endothelial cells and disseminates within the host.

Your feedback has been instrumental in guiding us towards enhancing the quality of our work. We are committed to incorporating these changes and presenting more comprehensive and clear results to address the potential confounding factors and strengthen the validity of our findings in the revised manuscript.

Best regards

**Part II – Major Issues: Key Experiments Required for Acceptance**
Please use this section to detail the key new experiments or modifications of existing experiments that should be absolutely required to validate study conclusions.

Reviewer #1: Major Comments:

1. In this study, to investigate the effect of Tp on microvascular endothelial cells, HMEC-1 cells were co-culture with Tp in vitro to simulate the infection environment of syphilis. However, to our knowledge, the technology of Tp in vitro culture is not yet mature. How can you ensure that Tp can play a role similar to in vivo infection during co-culture with HMEC-1 cells. In addition, although Tp can survive in vitro for a short time, whether it has corresponding infective activity remains unproven.

RESPONSE:

Thank you very much for your insightful comments. We appreciate your concerns and would like to address them as follows:

(a) Previous studies have revealed that *Tp* can enter the bloodstream of rabbits or traverse the *in vitro* human umbilical vein endothelial cell barrier within hours^[1-3]^, which is consistent with our findings (Fig 3D-E & 5A-B), collectively indicating that the role of *Tp* on penetrating the vascular endothelial barrier likely remains consistent in both *in vivo* and *in vitro* experiments. Despite the immaturity of *Tp*'s in vitro culture technology, we mitigated this limitation by focusing on short-term experiments (0 hours, 0.5 hours, 1 hour, 3 hours, and 6 hours of *Tp* infection). It is noteworthy that within the scientific community, experiments conducted within 24 hours are commonly considered short-term for studying syphilis infection and endothelial dysfunction^[4,5]^. Our results suggested that within the short co-culture period, *Tp* retained its capacity to interact with endothelial cells in a manner consistent with early stages of infection.

(b) In our previous study^[6]^, we found that the adhesive ability of G1-*Tp* (Generation 1, also utilized in this study) exhibited an increasing trend within 48 hours of co-incubation with cells under the same culture conditions *in vitro* (published data). This suggests that *Tp* can sustain perfect activity within 48 hours of in vitro culture.

(c) We prepared cell coverslips in a 24-well plate, seeded HMEC-1 cells onto the coverslips, and allowed them to adhere for 24 hours. Subsequently, live *Tp* (MOI = 2) was added to co-culture with HMEC-1 cells for durations of 6 and 8 hours. After the specified incubation times, the coverslips were removed, and *Tp* mobility was observed using darkfield microscopy to capture both videos and photos. Mobile *Tp* organisms were clearly visible (indicated by red arrows in S1A-B Fig), and corresponding videos were uploaded as S1 File in the submission system. Moreover, intradermal injection of *Tp* isolated from the 6-hour co-culture medium (Ltp group) successfully induced primary syphilitic skin lesions in a rabbit model (S1C Fig), demonstrating that *Tp* retained its infective activity. Conversely, *Tp* treated with antibiotics did not induce similar lesions (S1D Fig), affirming the importance of *Tp* 's viability in pathogenesis.

We hope these explanations address your concerns and underscore our dedication to advancing knowledge on *Tp*'s interactions with endothelial cells.

**S1 Fig. The viability and mortality of *Tp*. (A-B)** Motile *Tp* after stimulating HMEC-1 cells for (A) 6 hours and (B) 8 hours; observed by DFM under a 400-fold magnification, scale bar = 100 μm. **(C)** Rabbit back skin, 4 weeks after intradermal injection of live *Tp* isolated from the 6-hour co-culture medium. **(D)** Rabbit back skin, 4 weeks after intradermal injection of dead *Tp* inactivated by the co-cultured medium containing 1% (v/v) penicillin-streptomycin.

References of this response:

1.	RAIZISS GW, SEVERAC M. RAPIDITY WITH WHICH SPIROCHAETA PALLIDA INVADES THE BLOOD STREAM. Archives of Dermatology and Syphilology. 1937;35(6):1101-9. doi.org/10.1001/archderm.1937.01470240093008

2.	Cumberland MC, Turner TB. The rate of multiplication of Treponema pallidum in normal and immune rabbits. American journal of syphilis, gonorrhea, and venereal diseases. 1949;33(3):201-12. PMID:18121293.

3.	Lithgow KV, Tsao E, Schovanek E, Gomez A, Swayne LA, Cameron CE. Treponema pallidum Disrupts VE-Cadherin Intercellular Junctions and Traverses Endothelial Barriers Using a Cholesterol-Dependent Mechanism. Frontiers in microbiology. 2021;12:691731. doi.org/10.3389/fmicb.2021.691731 PMID:34354688.

4.	Wang YJ, Xu QY, Ye WM, Yi DY, Zheng XQ, Xie L, et al. Treponema pallidum Promotes the Polarization of M2 Subtype Macrophages to M1 Subtype Mediating the Apoptosis and Inhibiting the Angiogenesis of Human Umbilical Vein Endothelial Cells. ACS infectious diseases. 2023;9(12):2548-59. doi.org/10.1021/acsinfecdis.3c00401 PMID:37983134.

5.	Waugh S, Ranasinghe A, Gomez A, Houston S, Lithgow KV, Eshghi A, et al. Syphilis and the host: multi-omic analysis of host cellular responses to Treponema pallidum provides novel insight into syphilis pathogenesis. Frontiers in microbiology. 2023;14:1254342. doi.org/10.3389/fmicb.2023.1254342 PMID:37795301.

6.	Huang J, Jiang Y, Lin W, Chen R, Zhou J, Guo S, et al. Virulence and Adhesion of the Treponema pallidum Nichols Strain Simultaneously Decrease in a Continuous-Infection New Zealand White Rabbit Model. ACS infectious diseases. 2023;9(6):1221-31. doi.org/10.1021/acsinfecdis.2c00601 PMID:37192527.T

2. Different MOL of Tp were used for this study, but the specific quantitative method of Tp did not shown to be mentioned in the material method.

RESPONSE:

Thank you for bringing up this important point. In our study, we indeed utilized different multiplicities of infection (MOIs) of *Tp*, but the specific quantitative method for determining *Tp* concentration was not clearly outlined in the Materials and Methods section of the manuscript.

We apologize for this oversight and commit to providing a detailed explanation of the quantitative method for determining *Tp* concentration in the revised version of the manuscript. Here is the related text description:

**Preparation and enumeration of *Tp* Nichols strain**

The *Tp* Nichols strain was provided by Prof. Tiebing Zeng from University of South China. This strain was propagated with the rabbit infectivity test and resuspended to obtain live *Tp* suspension referred to the previous studies^[1,2]^. To obtain an inactivated *Tp* suspension, 1% (v/v) penicillin-streptomycin was added for a 30-minute incubation. The number of *Tp* organisms was determined using the dark field microscopy (DFM) enumeration method^[2,3]^:

A ten-microliter (10.0 μL) of *Tp* suspension was placed on a slide and covered with a 22.0 × 22.0 mm coverslip, providing an area of 4.84 cm^2^ (22.0 mm × 22.0 mm = 484 mm^2^ = 4.84 cm^2^) and a thickness of 0.0207 mm (10.0 μL = 0.01 cm^3^, 0.01 cm^3^/4.84 cm^2^ = 0.00207 cm = 0.0207 mm).

Spiral-shaped *Tp* organisms were counted in twenty to fifty of microscopic fields (40×) when each field contained more than 10 organisms; otherwise, at least one hundred fields were counted (recorded as X_1_, X_2_, …, X_n_, where n represented the number of fields).

In each field, the radius (r) of the DFM (Olympus, JAPAN; BX43) under the 40× microscope is 0.205 mm, resulting in a field area of 0.134 mm^2^ (πr^2^), and a volume of 2.73 × 10^-6^ cm^3^ (0.134 mm^2^ × 0.0207 mm = 2.73 × 10^-3^ mm^3^ = 2.73 × 10^-6^ cm^3^).

Therefore, the concentration (organisms/mL) was calculated using the formula: (X_1_ + X_2_ + … + X_n_)/(2.73 × 10^-6^ × n).

References of this response:

1.	Huang J, Jiang Y, Lin W, Chen R, Zhou J, Guo S, et al. Virulence and Adhesion of the Treponema pallidum Nichols Strain Simultaneously Decrease in a Continuous-Infection New Zealand White Rabbit Model. ACS infectious diseases. 2023;9(6):1221-31. doi.org/10.1021/acsinfecdis.2c00601 PMID:37192527.

2.	Lukehart SA, Marra CM. Isolation and laboratory maintenance of Treponema pallidum. Current protocols in microbiology. 2007;Chapter 12:Unit 12A.1. doi.org/10.1002/9780471729259.mc12a01s7 PMID:18770607.

3.	Chandler FW, Norins LC. Studies on the quantitation of intraocular Treponema pallidum. An in vitro model. The British journal of venereal diseases. 1969;45(2):117-20. doi.org/10.1136/sti.45.2.117

3. Result 4. There was no significant difference in PAKT1 level after Tp stimulation with MOL 1 and 2 in Fig4B. Why should the stimulation concentration with Mol 2 be used in subsequent experiments.

RESPONSE:

Thank you for your insightful comment regarding the pAKT1 levels after *Tp* stimulation in Figure 4B. Despite observing no significant difference in pAKT1 levels between MOI 1 and MOI 2, we chose to use an MOI of 2 in subsequent experiments based on the following considerations:

(a) Higher MOI values are likely to induce more consistent cellular responses in terms of downstream signaling pathways. In our study, when MOI value exceeded 1, a maximum plateau phase existed. To ensure sufficient phosphorylation of AKT1, the stimulation concentration with MOI 2 could fully guarantee its maximal kinase activity.

(b) Maintaining consistency with our previous experiments, including Western blotting for fibronectin and vimentin protein levels under *Tp* stimulation with varying conditions (Fig 1B, 1D, 1E, 1G, 2B, 2C, 2F, 2G, and 3B), immunofluorescence assays (Fig 1A, 2D, and 3C), and endothelial barrier traversal experiments (Fig 3D and 5A), necessitated the use of MOI 2. Furthermore, an MOI of 2 provided a robust and reproducible experimental condition for studying mechanisms such as fibronectin matrix disaggregation (Fig 1F) and vimentin phosphorylation (Fig 2E).

We appreciate your attention to this detail and will clarify this rationale in the revised manuscript to provide better context for our experimental choices. Thank you once again for your valuable feedback.

4. The Figure of Wb in Fig4F is not clear, please replace it with a clearer Figure.

RESPONSE:

Your attention to detail is greatly appreciated. We acknowledge the unclear quality of the Western blotting image regarding Fig 4F and have promptly replaced it with a clearer figure in the revised manuscript to ensure better visualization and interpretation of the data.

It's possible that the submission system automatically compresses images and reduces their resolution when generating the Manuscript Draft. Therefore, we have uploaded clearer and newly replaced images separately. By clicking the link in the upper right corner of the corresponding figure in the draft, you can also view a single high-definition image.

Once again, we apologize for any inconvenience caused.

5. Please detect the stability of cFN matrix and the spread of Tp infection after inhibition the phosphorylation of pSer39.

RESPONSE:

We are grateful for your thoughtful suggestion. Since we found that the phosphorylation of Ser39-vimentin (pSer39-vimentin) was the upstream of fibronectin (FN) matrix disaggregation, which subsequently facilitated the spread of *Tp* infection, we performed two kind of inhibition methods:

(a) We mutated the 39th serine to alanine (S39A) and overexpressed S39A-vimentin in HMEC-1 cells. Because alanine cannot be phosphorylated, HMEC-1 cells could not experience the phosphorylation of 39^th^ residue in vimentin, even under live *Tp* treatment. Hence, in our study, Western blotting analysis (Fig 3B) revealed that the level of insoluble FN (composing FN matrix) obviously increased, as well as the dense FN matrix observed in the immunofluorescence assay (Fig 3C). The endothelial barrier traversal experiment (Fig 3D & 3E) showed the enhanced capacity of HMEC-1 cells with S39A-vimentin overexpression (OE-S39A-Vim) for resisting *Tp* penetration, reflecting in a significant reduction in the number of *Tp* copies crossing into the lower chamber.

(b) We predicted the potential kinase of Ser39-vimentin and identified AKT kinase with both high score value and cut off value (Fig 4A). Here, we have further demonstrated the interaction between vimentin and AKT1 using co-immunoprecipitation (co-IP) experiments (S3 Fig), confirming a physical interaction between vimentin and AKT1. Therefore, we used the inhibitor agent of AKT to banish the pSer39-vimentin in HMEC-1. The Western blotting revealed an augment level of insoluble FN protein in the both live *Tp*/AKT inhibitor-treated group (Fig 4F), while the immunofluorescence assay (Fig 4G) verified a dense FN matrix around the cells in the same group, and fewer *Tp* cells were observed invading the HMEC-1 cells.

Taken together, these results provide substantial evidence that supports our findings and effectively address your question about FN matrix disaggregation and the spread of *Tp* infection after inhibition the phosphorylation of Ser39-vimentin. We believe these experiments enhance the robustness of our study. Thank you once again for your valuable feedback.

**S3 Fig. Co-immunoprecipitation of vimentin and AKT1.** Cell lysates from negative control and live *Tp*-infected HMEC-1 cells after a 6-hour infection period were subjected to immunoprecipitation using an anti-vimentin antibody **(A)** or an anti-AKT1 antibody **(B)**, followed by Western blotting to detect the immunoprecipitated complexes of AKT1 and vimentin. The IgG antibody was used to demonstrate the absence of non-specific binding, and the input lysates (10% of the total lysate) were probed to confirm the presence of both proteins in the lysates. Ctrl: negative control; Ltp: live *Tp*; IP: immunoprecipitation; IB: immunoblotting; Vim: vimentin.

6. Result 6. Is direct injection of cFN via intradermal injection similar to vaccination? How to ensure the functional structure of the protein *in vivo* after injected directly without any adjuvants? In addition, The PCR results in different tissues showed that the load of Tp in the PBS+Ltp group was also not high, Whether this indicates that Tp does not diffuse effectively *in vivo*, which will directly affect the reliability of this result.

RESPONSE:

We really value your insightful questions regarding Result 6. We have conducted further investigations and explanations to address the issues you raised.

Ⅰ. Direct injection of cFN (cellular fibronectin) via intradermal injection:

You inquired whether the direct injection of cFN via intradermal injection is similar to vaccination and how we ensure the functional structure of the protein *in vivo* without any adjuvants.

We understand the importance of this comparison. While intradermal injection is indeed a method used in vaccinations, the key difference in our study lies in the nature and purpose of the injected substance. In our study, cFN was administered as a modulating agent aimed at changing the microenvironment around microvascular endothelial cells and maintaining steady state of FN matrix in ECM, rather than as a traditional vaccine antigen.

(a) cFN protein purity and stability: The dry powder of cFN protein purchased from Sigma-Aldrich was highly purified and stabilized through rigorous preparation protocols to maintain its functional integrity. Prior to its use in the *in vivo* experiment, the protein's functionality was validated in *in vitro* tests (Fig 5), confirming that the protein exhibited expected biological activity, thereby ensuring its suitability for our study. And in our *in vivo* experiment, we dissolved the cFN dry powder with sterile PBS and immediately performed intradermal injection.

(b) Injection technique and dose: Intradermal injection, by its nature, deposits the protein into the dermal layer, where it is less likely to encounter harsh enzymatic degradation compared to other routes. Compared with studies involving proteins that potentially induce immunoprotective effects in *Tp*-challenged rabbits, a relatively low dose (2.5μg/site, 8 sites; 20μg in total) of cFN protein without any adjuvant was used in our study, unlikely to cause an immune response. For example, Lithgow et al. investigated the define syphilis vaccine candidate (Tp0751) by utilized Tp0751-immunized rabbits^[1]^, during which each rabbit was “sedated and immunized three separate times with water in oil emulsions made up of 0.52 mg mL^−1^ Tp0751 in aqueous buffer with TiterMax Gold adjuvant (Sigma, St Louis, MO, USA) in a 1:1 ratio. Each immunization was delivered as four 0.1 ml subcutaneous injections into the shoulders and two 0.04 ml intramuscular injections into the quadricep muscles, according to the manufacturer’s instructions”. We could calculate that the dose of immunized protein was (0.52 mg/mL × 0.1 ml × 4 + 0.52 mg/mL × 0.04 ml × 2) × 3 = 0.7488 mg = 748.8 μg, which is almost 40 times than the used dose in our study. And Delgado et al also performed a similar large dose of 200μg^[2]^.

(c) As shown in S2 File, there is a fairly high homology between human and rabbit FN proteins, including both the whole protein sequences and each domain. Furthermore, as we observed, the rabbits which only received cFN or pFN or laminin injection without Tp infection did not develop any inflammatory signs in the skin from Day1 to Day26 post-injection (S5A Fig). Besides, compared to the healthy rabbit, there is also no detective signal (through the ELISA assay) of anti-FN antibody in the peripheral blood of the FN-injected rabbits after 26 days post-intradermally injection.

Ⅱ. PCR results and *Tp* diffusion:

You noted that the PCR results showed a low load of *Tp* in different tissues in the PBS+Ltp group, raising concerns about the effective diffusion of *Tp* *in vivo* and the reliability of the results. We have taken this concern seriously and conducted further discussion in more detail:

(a) We want to explain why we euthanized the rabbits on Day 26. This decision was based on regarding the positive control group (PBS+Ltp group) as a baseline. Reliable evidence indicates that the production of TRUST antibodies is primarily due to cardiolipin released from damaged host cells^[3]^. Besides, our other study has demonstrated that after *Tp* stimulates vascular endothelial cells, the cardiolipin released from mitochondria into the cytoplasm can directly cause the TRUST titer to turn positive in rabbits even without *Tp* infection^[4]^. To ensure that the effects on the various tissues of the rabbits were due solely to *Tp* infection and to minimize the impact of immune and inflammatory responses on these tissues, we decided to euthanize the rabbits immediately when the TRUST titer in the PBS+Ltp group (Day26) turned positive (Table 2).

(b) It seemed that the *Tp* load in the PBS+Ltp group appeared low, but it should be noted that the *Tp* load of each rabbit we obtained corresponded to 20.0 mg per biopsy of skin (2 biopsies per rabbit), 20.0 mg of liver (2 biopsies per rabbit), 10.0 mg of spleen (2 biopsies per rabbit), and one entire lymph node (about 5.0 mm long and weighs 25.0 mg) from the popliteal fossa. Due to each organ being biopsied for qPCR detection of *Tp* load with only about 10 mg to 25 mg, the volume of these organs is actually much larger, especially the liver and spleen. For example, the liver weighs approximately 70 grams per rabbit, and the spleen weighs about 2 grams per rabbit. Since biopsies were taken randomly from distant locations within the same organ (one close to the centrally vascularized area and one near the edge), if the *Tp* loads corresponding to each biopsy was extrapolated to the entire organ by weight, the overall *Tp* load in each organ would be much higher, potentially hundreds to thousands of times the values shown in the Fig6. Moreover, Furthermore, during *Tp* infection in rabbits, we administered an intradermal injection with a *Tp* concentration of 10^6^/mL, with 0.1 mL per injection site, across 8 sites, resulting in a total of just under 10^6^ *Tp* organisms. We believe that the dissemination level of *Tp* in the liver and spleen of the PBS+Ltp group by Day26 post-infection was actually not low; and the decreasing trend observed in the cFN+Ltp group was more significant, as evidenced by the reduced *Tp* load, the later appearance of specific treponemal antibodies (TPPA) in the peripheral blood, and the lowest TPPA titers observed on Day 26 (Table 1).

In addition, since *Tp* may infect various systems and organs, and its organ phagocytosis and diffusion are still unknown, we will include more organs for further study to verify the uptake of *Tp* within different organisms and continuously monitor the change of its load in the most phagocytic organs.

We hope these clarifications address your concerns and enhance the robustness of our study. Thank you once again for your expert opinion and suggestions.

References of this response:

1.	Lithgow KV, Hof R, Wetherell C, Phillips D, Houston S, Cameron CE. A defined syphilis vaccine candidate inhibits dissemination of Treponema pallidum subspecies pallidum. Nature communications. 2017;8(1):14273. doi.org/10.1038/ncomms14273

2.	Delgado KN, Montezuma-Rusca JM, Orbe IC, Caimano MJ, La Vake CJ, Luthra A, et al. Extracellular Loops of the Treponema pallidum FadL Orthologs TP0856 and TP0858 Elicit IgG Antibodies and IgG(+)-Specific B-Cells in the Rabbit Model of Experimental Syphilis. mBio. 2022;13(4):e0163922. doi.org/10.1128/mbio.01639-22 PMID:35862766.

3.	Gao K, Shen X, Lin Y, Zhu XZ, Lin LR, Tong ML, et al. Origin of Nontreponemal Antibodies During Treponema pallidum Infection: Evidence From a Rabbit Model. The Journal of infectious diseases. 2018;218(5):835-43. doi.org/10.1093/infdis/jiy241 PMID:29701849.

3.	Luo X, Xie X, Zhang L, Shi Y, Fu B, Yuan L, et al. Uncovering the mechanisms of host mitochondrial cardiolipin release in syphilis: Insights from human microvascular endothelial cells. International Journal of Medical Microbiology. 2024;316:151627. doi.org/10.1016/j.ijmm.2024.151627

Reviewer #2: These studies were carried out in human HMEC-1 cells. A key experiment that seems missing is to determine how vimentin-null HMEC-1 cells respond to Tp infection. The authors could then determine how the virus-driven over-expression of wild type vimentin or S39A vimentin (in a vimentin null background) influences the patterns of gene expression and the Tp effect on fibronectin/ECM. In a similar way, it would be interesting to know how living and dead Tp influence patterns of gene expression.

Generating Vim-/- HMEC-1 cell lines should be a straightforward using CRISPR-Cas9; their immunofluorescence microscopy assay would enable them to quickly establish whether it is vimentin or the disruption of normal vimentin organization, in response to AKT-1 (e.g. by VimS39A expression) that is involved in the cell's response to Tp.

I am not commenting on the rabbit experiments, beyond my expertise.

RESPONSE:

Thank you for your helpful insights and recommendations. We appreciate your thorough review and the valuable points you raised.

Vimentin is distributed within the cytoplasm, on the cell membrane, and outside the cell, playing crucial roles in coordinating morphology, transduction, signaling pathways, motility, and inflammatory responses^[1-3].^ There have been no reports in the literature indicating that complete loss of vimentin in HMEC-1 cells affects their viability. Although vimentin null mice can survive, numerous studies have shown that these mice exhibit vascular system malformations and compromised endothelial barrier function (characterized by vascular leakage and developmental abnormalities), further manifesting that the loss of vimentin impacts cellular mechanosensing, stiffness, and acto-myosin contractility^[4-7]^.

Our study focused on the changes in vimentin as a cytoskeletal protein in the cytoplasm following *Tp* infection. We considered that constructing vimentin null (Vim^-/-^) HMEC-1 cell using CRISPR-Cas9 could affect various aspects of HMEC-1 cell during *in vitro* culture, beyond merely the inability to form a cytoskeleton in the cytoplasm and thus failure to maintain the stability of the integrin-FN matrix.

We agree that investigating the response of Vim^-/-^ HMEC-1 cells to *Tp* infection would provide critical insights. However, during our preliminary attempts to generate Vim^-/-^ HMEC-1 cells using CRISPR-Cas9, we encountered significant challenges. Post-antibiotic selection, the viability of the remaining cells was less than 1%, and they adopted more circular shape. The few cells that survived did not successfully proliferate during single-cell cloning. Therefore, we speculated that complete vimentin knockout in HMEC-1 cells might necessitate extensive optimization. Considering the technical challenges and time constraints, we opted to use siRNA interference to reduce vimentin expression, as well as fibronectin expression. This approach allowed us to study the effects of vimentin reduction on *Tp* infection without the immediate complications of complete knockout.

FN matrix disaggregation refers to changes in the matrix structure from dense to sparse without altering the total protein content of monomers or dimers assembled into the matrix, while the lack of FN would lead to the almost disappearance of the matrix (S6 Fig). Inhibited expression of vimentin protein in HMEC-1 cells by siRNA (HMEC-1^siVim^) slightly reduced the protein level of fibronectin (S7A Fig), indicating that vimentin knockdown is unlikely to affect FN synthesis. However, the total fibronectin significantly decreased in HMEC-1^siVim^ after *Tp* infection (S7B Fig), along with a sparser fibronectin matrix around HMEC-1^siVim^ cells observed by the immunofluorescence assay (S7C Fig), which was different from the results in Fig 1B-D. After live *Tp* infection, the total FN levels in HMEC-1 wild-type cells did not change, suggesting that live *Tp* did not induce FN protein degradation or synthesis under the condition of enough vimentin expression. However, knockdown of vimentin reduced the total protein levels of various forms of vimentin, including the vimentin reserve that binds to integrins on the cell membrane to stabilize extracellular FN, which may disrupt the FN matrix by enhancing both the matrix transformation pathway and the FN degradation pathway during *Tp* infection, further illustrating the close relationship between vimentin and FN.

In conclusion, while we acknowledge the importance of generating vimentin-null HMEC-1 cells, the technical challenges we encountered led us to employ siRNA interference as a practical alternative. We believe this approach provided valuable insights into the role of vimentin in the context of our study and laid the groundwork for future studies involving the complete vimentin knockout model.

**S6 Fig. Western blotting and immunofluorescence analysis of HMEC-1 cells with fibronectin knockdown by siRNA. (A)** Protein levels of total fibronectin in HMEC-1 cells with fibronectin knockdown by siRNA (HMEC-1^siFN^) and cells with scramble siRNA interference as negative control (HMEC-1^siNC^), respectively. **(B)** Protein levels of total fibronectin in HMEC-1 ^siFN^ and HMEC-1^siNC^ co-cultured with live *Tp* (MOI 2) for 6 hours, respectively. **(C)** Fibronectin matrix of HMEC-1^siFN^ and HMEC-1^siNC^ co-cultured with live *Tp* (MOI 2) for 6 hours, respectively; observed by fluorescence microscopy, blue for the nucleus, green for the FN matrix, red for *Tp*, scale bar = 100 μm. NC: negative control; FN: fibronectin; Ltp: live *Tp*.

**S7 Fig. Western blotting and** **immunofluorescence analysis of HMEC-1 cells with vimentin knockdown by siRNA. (A)** Protein levels of pSer39-vimentin, total vimentin, and total fibronectin in HMEC-1 cells with vimentin knockdown by siRNA (HMEC-1^siVim^) and cells with scramble siRNA interference as negative control (HMEC-1^siNC^), respectively. **(B)** Protein levels of pSer39-vimentin, total vimentin, and total fibronectin in HMEC-1^siVim^ and HMEC-1^siNC^ that co-cultured with live *Tp* (MOI 2) for 6 hours, respectively. **(C)** Fibronectin matrix of HMEC-1^siVim^ and HMEC-1^siNC^ that co-cultured with live *Tp* (MOI 2) for 6 hours, respectively; as observed by fluorescence microscopy; blue for the nucleus, green for the FN matrix, and red for *Tp*, scale bar = 100 μm.. NC: negative control; Vim: vimentin; FN: fibronectin; Ltp: live *Tp*.

References of this response:

1.	Swoger M, Gupta S, Charrier EE, Bates M, Hehnly H, Patteson AE. Vimentin Intermediate Filaments Mediate Cell Morphology on Viscoelastic Substrates. ACS applied bio materials. 2022;5(2):552-61. doi.org/10.1021/acsabm.1c01046 PMID:34995457.

2.	Ridge KM, Eriksson JE, Pekny M, Goldman RD. Roles of vimentin in health and disease. Genes & development. 2022;36(7-8):391-407. doi.org/10.1101/gad.349358.122 PMID:35487686.

3.	Mendez MG, Kojima S, Goldman RD. Vimentin induces changes in cell shape, motility, and adhesion during the epithelial to mesenchymal transition. FASEB journal : official publication of the Federation of American Societies for Experimental Biology. 2010;24(6):1838-51. doi.org/10.1096/fj.09-151639 PMID:20097873.

4.	Nieminen M, Henttinen T, Merinen M, Marttila–Ichihara F, Eriksson JE, Jalkanen S. Vimentin function in lymphocyte adhesion and transcellular migration. Nature Cell Biology. 2006;8(2):156-62. doi.org/10.1038/ncb1355

5.	Antfolk D, Sjöqvist M, Cheng F, Isoniemi K, Duran CL, Rivero-Muller A, et al. Selective regulation of Notch ligands during angiogenesis is mediated by vimentin. Proceedings of the National Academy of Sciences. 2017;114(23):E4574-E81. doi.org/doi:10.1073/pnas.1703057114

6.	Shen Y, Wu H, Lu PJ, Wang D, Shayegan M, Li H, et al. Effects of Vimentin Intermediate Filaments on the Structure and Dynamics of In Vitro Multicomponent Interpenetrating Cytoskeletal Networks. Physical Review Letters. 2021;127(10):108101. doi.org/10.1103/PhysRevLett.127.108101

7.	Langlois B, Belozertseva E, Parlakian A, Bourhim M, Gao-Li J, Blanc J, et al. Vimentin knockout results in increased expression of sub-endothelial basement membrane components and carotid stiffness in mice. Scientific Reports. 2017;7(1):11628. doi.org/10.1038/s41598-017-12024-z

Reviewer #3: 1. The authors employed both live and deceased Tp in the study, did there any evidence supportting the viability and mortality of Tp.

RESPONSE:

Thank you for your careful consideration and comments regarding the viability and mortality of *Tp* used in our study. We have provided additional evidence to support the viability and mortality of *Tp*.

Researchers in the field of syphilis investigation typically consider experiments conducted within 24 hours as short-term experiments for studying *Tp* infection and endothelial dysfunction^[1,2]^. And the experiments conducted in our study focused on short-term *Tp* infection (for 0 hours, 0.5 hours, 1 hours, 3 hours, and 6 hours), suggesting *Tp* retained its capacity of great viability and mortality interacting with endothelial cells during the co-culture period.

In our previous study^[3]^, we found that the adhesive ability of G1-*Tp* (Generation 1, also utilized in this study) exhibited an increasing trend within 48 hours of co-incubation (under the same culture conditions in this study) with cells *in vitro* (published data), indicating that *Tp* could sustain its activity within 48 hours of culture *in vitro*.

Specifically, we have included an additional observation of *Tp* under dark field microscopy, revealing that mobile *Tp* could be observed after the co-culture with HMEC-1 cells for 6 hours and even 8 hours (red arrows in the S1A-B Fig indicated *Tp*, which were still active in the corresponding videos we uploaded in the submission system as S1 File). Besides, intradermal injection of *Tp* separated from the 6-hour co-cultured medium (the Ltp group) could successfully establish a rabbit model with primary syphilitic skin lesion (S1C Fig), demonstrating a remained corresponding infective activity of *Tp*, while the dead Tp underwent anti-biotics killing could not (S1D Fig).

We hope these explanations address your concerns and could help us to improve the rigor and clarity of our data.

**S1 Fig. The viability and mortality of *Tp*. (A-B)** Motile *Tp* after stimulating HMEC-1 cells for (A) 6 hours and (B) 8 hours; observed by DFM under a 400-fold magnification, scale bar = 100 μm. **(C)** Rabbit back skin, 4 weeks after intradermal injection of live *Tp* isolated from the 6-hour co-culture medium. **(D)** Rabbit back skin, 4 weeks after intradermal injection of dead *Tp* inactivated by the co-cultured medium containing 1% (v/v) penicillin-streptomycin.

References of this response:

1.	Kao WA, Pětrošová H, Ebady R, Lithgow KV, Rojas P, Zhang Y, et al. Identification of Tp0751 (Pallilysin) as a Treponema pallidum Vascular Adhesin by Heterologous Expression in the Lyme disease Spirochete. Sci Rep. 2017;7(1):1538. doi.org/10.1038/s41598-017-01589-4 PMID:28484210.

2.	Parker ML, Houston S, Pětrošová H, Lithgow KV, Hof R, Wetherell C, et al. The Structure of Treponema pallidum Tp0751 (Pallilysin) Reveals a Non-canonical Lipocalin Fold That Mediates Adhesion to Extracellular Matrix Components and Interactions with Host Cells. PLoS pathogens. 2016;12(9):e1005919. doi.org/10.1371/journal.ppat.1005919 PMID:27683203.

3.	Huang J, Jiang Y, Lin W, Chen R, Zhou J, Guo S, et al. Virulence and Adhesion of the Treponema pallidum Nichols Strain Simultaneously Decrease in a Continuous-Infection New Zealand White Rabbit Model. ACS infectious diseases. 2023;9(6):1221-31. doi.org/10.1021/acsinfecdis.2c00601 PMID:37192527.

Reviewer #4: 1. Most of the conclusions were made based on the correlation of two different phenotypes. The examples are as follows:

(1) (line 283-310), The results showed the correlation of the levels of Vimentin with the levels of cFN. Such correlations do not necessarily suggest “TP-induced vimentin phosphorylation and rearrangement promoted disaggregation if cFN” concluded in the line 281-282. To directly tie vimentin into the contributor of the cFN pathways, a vimentin-knock down cells would need to be added in the same experimental setup of infection with Ltp.

RESPONSE:

We value your constructive criticism and suggestions regarding the relationship between the level of vimentin and that of fibronectin (FN). We have provided additional explanations and experiments to support the conclusion of “*Tp*-induced vimentin phosphorylation and rearrangement promoted disaggregation of the cFN matrix” as stated in lines 281-282 of the original manuscript.

(a) In Fig 2A-C, we analyzed the protein levels of total vimentin in HMEC-1 cells infected with live *Tp* under different conditions: varying MOIs (Fig 2A), different infection durations (Fig 2B), and live vs. dead *Tp* (Fig 2C). Our results show that the total vimentin levels remained unchanged across these conditions and were independent of *Tp* viability. However, live *Tp* infection led to a decrease in insoluble vimentin and an increase in soluble vimentin with increasing MOIs and prolonged infection times. In Fig 2D, we observed an accumulation of soluble vimentin around the nucleus in the Ltp group. Consistent with the previous study^[1]^, we demonstrated significant phosphorylation of the 39^th^ serine residue of vimentin (Ser39-vimentin) (Fig 2E-G), which increased its solubility.

(b) Given that *Tp* infection did not affect total vimentin levels but enhanced its post-translational modification, we aimed to inhibit this modification without reducing total vimentin protein levels. We constructed a vimentin expression plasmid with a Ser39-to-alanine (S39A) mutation, preventing phosphorylation at this site (Fig 3A). This mutation-maintained vimentin in its insoluble form, preserving normal cytoskeletal morphology (which was also demonstrated by the previous study^[1]^), rescued the decrease in insoluble fibronectin necessary for FN matrix assembly (Fig 3B), prevented cFN matrix disaggregation (Fig 3C), and reduced *Tp*’s ability to cross the HMEC-1 monolayer (Fig 3E).

Combining findings from (a) and (b), we believe that inhibiting Ser39-vimentin phosphorylation at the post-translational level directly highlights the critical role of this site in mediating FN matrix disaggregation under *Tp* infection.

On the other hand, we appreciate your constructive suggestion regarding the changes in the FN matrix after the knockdown of vimentin. Based on your feedback, we have conducted supplementary experiments into the revised manuscript, providing stronger evidence for our conclusions.

(c) As shown in S7 Fig, we revealed that the knockdown of vimentin in HMEC-1 cells by siRNA (HMEC-1^siVim^) induced a slight decrease in total FN (S7A Fig), indicating that vimentin knockdown is unlikely to affect FN synthesis. Surprisingly, after *Tp* infection, HMEC-1^siVim^ cells exhibited a significant decrease in total FN (S7B Fig). The immunofluorescence assay revealed that *Tp* affected the ECM, resulting in a sparser FN matrix around HMEC-1^siVim^ cells (S7C Fig). In contrast, the total FN levels in HMEC-1 wild-type cells (HMEC-1^WT^) with enough vimentin expression, did not change after live *Tp* infection (Fig 1B-D), suggesting that live *Tp* did not induce FN protein degradation or synthesis. However, in HMEC-1^siVim^ cells, *Tp* may disrupt the FN matrix by enhancing both the matrix transformation pathway and the FN degradation pathway, further illustrating the close relationship between vimentin and FN.

Taken together, *Tp* infection alone did not lead to changes in the total levels of FN protein or vimentin protein in vascular endothelial cells. However, phosphorylation of Ser39-Vim significantly reduced FN protein insolubility and caused FN matrix disaggregation (disaggregation refers to changes in the matrix structure from dense to sparse without altering the total protein content of monomers or dimers assembled into the matrix). Knockdown of vimentin reduced the total protein levels of various forms of vimentin, including the vimentin reserve that binds to integrins on the cell membrane to stabilize extracellular FN, likely leading to an enhanced FN degradation trend. Further *Tp* infection of vimentin knockdown endothelial cells severely interfered with the FN matrix, affecting both the density of the structure and the levels of FN monomers assembled into the matrix.

We hope that these explanations and results demonstrate the critical role of vimentin in the cFN pathway and provide a clearer understanding of the relationship between vimentin and FN in the context of *Tp* infection.

**S7 Fig. Western blotting and immunofluorescence analysis of HMEC-1 cells with vimentin knockdown by siRNA. (A)** Protein levels of pSer39-vimentin, total vimentin, and total fibronectin in HMEC-1 cells with vimentin knockdown by siRNA (HMEC-1^siVim^) and cells with scramble siRNA interference as negative control (HMEC-1^siNC^), respectively. **(B)** Protein levels of pSer39-vimentin, total vimentin, and total fibronectin in HMEC-1^siVim^ and HMEC-1^siNC^ that co-cultured with live *Tp* (MOI 2) for 6 hours, respectively. **(C)** Fibronectin matrix of HMEC-1^siVim^ and HMEC-1^siNC^ that co-cultured with live *Tp* (MOI 2) for 6 hours, respectively; as observed by fluorescence microscopy; blue for the nucleus, green for the FN matrix, and red for *Tp*, scale bar = 100 μm.. NC: negative control; Vim: vimentin; FN: fibronectin; Ltp: live *Tp*.

References of this response:

1.	Kim J, Jang J, Yang C, Kim EJ, Jung H, Kim C. Vimentin filament controls integrin α5β1-mediated cell adhesion by binding to integrin through its Ser38 residue. FEBS letters. 2016;590(20):3517-25. doi.org/10.1002/1873-3468.12430 PMID:27658040.

(2) (line 311-328), These results showed the correlation of the levels of AKT1 and cFN. Such correlations do not necessarily suggest “Tp facilitated the phosphorylation of Ser39-vimentin via pAKT1 pathway” listed in line 311. AKT1 needs to be knocked down in the cells to directly tie AKT1 to cFN.

RESPONSE:

Thank you for your insightful comments. We have carefully considered your suggestion regarding the need to knock down AKT1 to directly establish its role in the correlation between AKT1 levels and cFN.

(a) We believe our existing data have thoroughly demonstrated that AKT1 is indeed the kinase responsible for phosphorylating Ser39-vimentin. This supports our conclusion that "*Tp* facilitated the phosphorylation of Ser39-vimentin via the pAKT1 pathway." Below is the detailed explanation from our original manuscript:

Our findings obtained from online prediction system indicated that the Ser39 residue is most likely phosphorylated by AGC kinases (Fig 4A), aligning with previous research suggesting that pSer39-vimentin can be mediated by AKT1^[1]^. To validate this, we measured phosphorylated AKT1 (pAKT1) levels in HMEC-1 cells and found increased pAKT1 with higher MOIs or longer durations (Fig 4B-C). The Ltp group showed significantly higher pAKT1 levels compared to other groups, with total AKT1 expression remaining unchanged (Fig 4D). Pre-treatment with the AKT1 inhibitor Capivasertib reversed the *Tp*-induced upregulation of pAKT1, increased insoluble cFN, and inhibited soluble cFN (Fig 4E-F). The Ltp+Capivasertib group did not show cFN matrix disaggregation (Fig 4G), unlike the Ltp group, indicating that reduced AKT1 activity prevented *Tp*-induced pSer39-vimentin and cFN matrix disaggregation.

(b) To address your concerns, we have conducted additional experiments where AKT1 was knocked down in the cells. The results from these experiments have been integrated with the existing data:

In Fig 4E, we demonstrated that total vimentin levels remained unchanged, while pSer39-vimentin decreased during *Tp* infection, indicating that AKT1 did not affect vimentin synthesis or degradation but impacted vimentin phosphorylation following live *Tp* stimulation. Notably, knocking down AKT1in HMEC-1 (HMEC-1^siAKT1^) cells reduced total FN levels, while total vimentin remained unchanged, even significantly in the absence of *Tp* stimulation (S8A Fig). This finding is consistent with previous literatures^[2,3]^, which suggests that AKT1 protein levels influence total FN through a pathway independent of vimentin, rather than via vimentin phosphorylation (i.e., AKT1 kinase activity does not affect total FN protein levels). Moreover, HMEC-1^siAKT1^ cells showed a more pronounced decrease in total FN upon stimulation with live *Tp* (S8B Fig). Mechanistically, there may be a compensatory balance to some extent, corresponding to a dual effect: i) AKT1 reduction causing decreased total FN (negative regulation) and ii) AKT1 reduction leading to decreased pSer39-vimentin and thereby increased the stability of FN matrix (positive regulation), resulting in a compensatory balance. As a result, even though the total amount of FN was significantly reduced, the cord-like aggregates were still partially present (S8C Fig).

These results underscore the critical role of AKT1 in mediating protein degradation, matrix formation, and matrix disassembly of FN, and they provide further support for the hypothesis that *Tp* facilitates the phosphorylation of Ser39-vimentin via the pAKT1 pathway.

**S8 Fig. Western blotting and immunofluorescence analysis of HMEC-1 cells with AKT1 knockdown by siRNA. (A)** Protein levels of AKT1, vimentin, and fibronectin in HMEC-1 cells with AKT1 knockdown by siRNA (HMEC-1^siAKT1^) and cells with scramble siRNA interference as negative control (HMEC-1^siNC^), respectively. **(B)** Protein levels of pSer39-vimentin, total vimentin, and fibronectin in HMEC-1 ^siAKT1^ and HMEC-1^siNC^ co-cultured with live *Tp* (MOI 2) for 6 hours, respectively. **(C)** Fibronectin matrix of HMEC-1 ^siAKT1^ and HMEC-1^siNC^ co-cultured with live *Tp* (MOI 2) for 6 hours, respectively; observed by fluorescence microscopy, blue for the nucleus, green for the FN matrix, red for *Tp*, scale bar = 100 μm. NC: negative control; Vim: vimentin; FN: fibronectin; Ltp: live *Tp*.

References of this response:

1.	Zhu QS, Rosenblatt K, Huang KL, Lahat G, Brobey R, Bolshakov S, et al. Vimentin is a novel AKT1 target mediating motility and invasion. Oncogene. 2011;30(4):457-70. doi.org/10.1038/onc.2010.421 PMID:20856200.

2.	Xin X, Chen S, Khan ZA, Chakrabarti S. Akt activation and augmented fibronectin production in hyperhexosemia. American journal of physiology Endocrinology and metabolism. 2007;293(4):E1036-44. doi.org/10.1152/ajpendo.00271.2007 PMID:17666488.

3.	White ES, Sagana RL, Booth AJ, Yan M, Cornett AM, Bloomheart CA, et al. Control of fibroblast fibronectin expression and alternative splicing via the PI3K/Akt/mTOR pathway. Experimental cell research. 2010;316(16):2644-53. doi.org/10.1016/j.yexcr.2010.06.028 PMID:20615404.

(3) (line 329-351) These results aimed to demonstrate the title in line 329 “Dense cFN matrix attenuated Tp penetration through the trans-endothelial barrier in vitro.” In order to make such conclusions, the experimental design should include the cells deficient of cFN (i.e., cFN-knockdown cells) and examine the Tp penetration. The current experimental design does not lead to such a conclusion.

RESPONSE:

We value your constructive suggestions. To investigate the impact of the FN matrix on TP penetration of the vascular endothelial barrier, there are typically two experimental approaches: upregulating cFN or reducing cFN. Given preliminary results indicating FN matrix sparsity, we prioritized adding additional cFN to observe its effect on TP infection.

However, we agree that including cFN-deficient cells should also be considered. We have taken your suggestion into account and conducted additional experiments using FN-knockdown HMEC-1 (HMEC-1^siFN^) cells to address this aspect. As shown in S6 Fig, upon infection of HMEC-1^siFN^ cells with live *Tp*, total FN protein levels rapidly decrease, which resulted in reduced FN matrix assembly and extensive disaggregation of existing matrix. During this process, we discovered that compared to knocking down AKT1 or vimentin, HMEC-1 cells with FN knockdown exhibited reduced proliferative activity. This is likely because the absence of FN leads to almost no FN aggregation in the ECM, preventing the formation of fibrous FN polymers, and the near-total disappearance of the FN matrix may hinder the cells from maintaining their biologically supported morphology. In this context, FN knockdown's overall impact on the cells might overshadow the regulation of TP infecting cells, potentially not achieving our desired observational goals.

Nonetheless, this positive attempt has provided us with valuable insights regarding the role of cFN in mediating *Tp* penetration through endothelial barriers. Thank you again for your insightful comments.

**S6 Fig. Western blotting and immunofluorescence analysis of HMEC-1 cells with fibronectin knockdown by siRNA. (A)** Protein levels of total fibronectin in HMEC-1 cells with fibronectin knockdown by siRNA (HMEC-1^siFN^) and cells with scramble siRNA interference as negative control (HMEC-1^siNC^), respectively. **(B)** Protein levels of total fibronectin in HMEC-1 ^siFN^ and HMEC-1^siNC^ co-cultured with live *Tp* (MOI 2) for 6 hours, respectively. **(C)** Fibronectin matrix of HMEC-1^siFN^ and HMEC-1^siNC^ co-cultured with live *Tp* (MOI 2) for 6 hours, respectively; observed by fluorescence microscopy, blue for the nucleus, green for the FN matrix, red for *Tp*, scale bar = 100 μm. NC: negative control; FN: fibronectin; Ltp: live *Tp*.

These weaknesses of experimental design are major, leading to the current results to be unsupportive for the conclusions the authors would like to make.

RESPONSE:

Thank you for your thorough review and valuable suggestions. Based on your proposed experimental plan, we have supplemented it as comprehensively as possible and have analyzed the corresponding results alongside those from the original manuscript. As a result, the conclusion that *Tp* mediates the pAKT1/pSer39-Vimentin/Fibronectin matrix pathway to promote its own trans-endothelial dissemination is now more reliable, having been further demonstrated through multiple experiments utilizing additional siRNA knockdown cell lines (HMEC-1^siVim^, HMEC-1^siAKT1^, and HMEC-1^siFN^).

We appreciate your comments, which have systematically clarified the pivotal roles of AKT1 and vimentin in promoting *Tp*-induced FN matrix disaggregation, integrating both supplementary and existing experimental findings.

2. In line 352 to 374, the authors attempted to pre-incubated cFN with T. pallidum and inoculate rabbits with cFN-T. pallidum mixtures and compared the resulting T. pallidum infectivity with the infectivity from the untreated T. pallidum. As there is always some unbound cFN in the mixtures of cFN and T. pallidum, the unbound cFN may have some physiological functions. Therefore, it is unclear whether the dissemination inhibition in cFN-treated T. pallidum is due to the binding of cFN to T. pallidum or the physiological functions caused by unbound cFN. pallidum. Therefore, such experimental design for the in vivo experiments introduced many confounding factors, which does not necessarily support the conclusion that “cFN inhibited the cutaneous in situ dissemination of Tp and its organic infection in vivo” in line 353.

RESPONSE:

Thank you for your insightful comments. We are dedicated to addressing these concerns in our revised manuscript by providing additional context to clarify the observed effects in our *in vivo* experiment. Here are the key points explained concisely:

(a) As shown in Fig 6A, we did not pre-mix proteins (cFN, pFN, or laminin) with *Tp* (which would emphasize protein-*Tp* adherence). Instead, proteins were injected intradermally into the backs of rabbits, followed by in situ injection of live *Tp* after 1 hour. This approach allowed for localized increase of proteins in the skin and mimics the established infection process of *Tp* crossing endothelial barriers post-local skin entry.

(b) Based on our *in vitro* findings, we concluded that *Tp* promoted FN matrix disaggregation independently of its binding to FN. Instead, this process was mediated through regulation of vimentin phosphorylation in endothelial cells. There was no evidence suggesting that *Tp* binding to FN affects vimentin phosphorylation levels.

(c) Developing genetically edited rabbits for animal experiments remains challenging. Future efforts will concentrate on creating rabbits with endothelial FN knockout specifically in skin vessels to reduce confounding factors and enhance the credibility of animal experimental results.

Thank you for your constructive feedback, which undoubtedly strengthens the rigor and clarity of our study's conclusions.

3. The model that the authors would like to test in this study is to tie fibronectin disaggregation triggered by T. pallidum to T. pallidum transmigration through microvascular endothelial cells. However, the other model that was also raised to explain the transmigration and vascular adhesion is that T. pallidum by using its surface protein to bind to fibronectin to promote such vascular interactions (PMID28484210, 27683203). Therefore, the dissemination phenotypes observed in vivo and the transmigration observed in trans-well can be caused by this adhesion model rather than the model the authors proposed. Before additional experiments differentiate the fibronectin disaggregation-mediated vs. fibronectin adhesion-mediated T. pallidum dissemination/transmigration, it is difficult to make the conclusion that these results support the title of this manuscript.

RESPONSE:

We appreciate your thoughtful comments and suggestions regarding our manuscript. Regarding your concern about the models proposed in our study, we acknowledge the alternative model suggesting that *Tp* may utilize its surface proteins to bind fibronectin, thereby promoting vascular interactions, as described in previous studies (PMID28484210, 27683203).

To clarify, our study primarily investigated the role of fibronectin disaggregation triggered by *Tp* and its correlation with *Tp* transmigration through microvascular endothelial cells. Additionally, we have conducted an additional experiment to assess the binding of *Tp* to cellular fibronectin (cFN), plasma fibronectin (pFN), and laminin, demonstrating that *Tp* exhibits maximal adherence to laminin instead of cFN (S4 Fig). Combining this finding with existing data, we speculate that the adherence to ECM protein by *Tp* may not directly relate to the cFN matrix disaggregation and consequent dissemination phenotypes.

On the other hand, previous studies (PMID28484210, 27683203) explored the interactions between *Tp* and host cells through heterologously expressing the TP0751 protein (a *Tp* surface protein) in *Borrelia burgdorferi*. This artificial intervention may differ from the natural infection of *Tp* in our experiments in several ways: i) There may be differences in the expression of Tp0751 protein, and whether the protein would exert the same adhesion properties under the conditions of heterologous expression and natural infection remains to be discussed. ii) These studies did not show that overexpression or inhibition of Tp0751 protein could trigger structural alterations in the FN matrix around host cells. iii) The adhesion of *Tp* to the ECM protein is not only through the TP0751 protein, but also the result of the joint action of many surface proteins. Taken together, we propose that fibronectin matrix disaggregation-mediated and fibronectin adhesion-mediated *Tp* dissemination/transmigration are actually two independent pathways, and of course, these two independent pathways can play a synergistic role in accelerating the spread of infection throughout the infection process.

We acknowledge the importance of differentiating between these mechanisms in future experiments to better elucidate the pathways involved in *Tp* dissemination and transmigration. As suggested, we are planning additional experiments to specifically differentiate between fibronectin disaggregation-mediated versus fibronectin adhesion-mediated *Tp* dissemination/transmigration. These experiments will provide a more comprehensive understanding and strengthen the conclusions drawn in our manuscript.

Thank you once again for your valuable feedback, which has prompted us to further refine our experimental approach and interpretations.

**S4 Fig.** **The adhesion capability of *Tp* to different ECM proteins.** **(A)** Nonadherent Tp (red arrow) in the supernatant combined with those washed down by PBS, after *Tp*’s adhesion to cFN, pFN, or laminin for 6 hours; observed by DFM under a 400-fold magnification, scale bar = 100 μm. **(B)** Quantification of non-adherent *Tp* per field of view under a 400-fold magnification using DFM. cFN: cellular fibronectin; pFN: plasma fibronectin; n.s.: no significance; ***: p value < 0.001.

**Part III – Minor Issues: Editorial and Data Presentation Modifications**
Please use this section for editorial suggestions as well as relatively minor modifications of existing data that would enhance clarity.

Reviewer #1: Minor Comments:

1. In the section of introduction, the description of research progresss in Fibronectin, extracellular matrix, and microvascular endothelial barrier is too simple and lacks logic, which makes it difficult to agree with the necessity of this study.

RESPONSE:

Thank you for your thorough and insightful review. We understand that the current description may appear too simplistic and lacking in logical flow, which might make it challenging to grasp the necessity of our study. In response to your valuable suggestion, we have undertaken the following steps in the Introduction Section of our revised manuscript:

(a) We have provided a more comprehensive overview of the current research and clearly linked existing research gaps to the objectives of our study. This aims to articulate the rationale for our research by demonstrating how our study addresses these gaps and contributes to the field.

(b) We have strengthened the justification for the necessity of our study by explaining how it builds on previous work and its potential impact on understanding and treating relevant conditions.

We value your constructive criticism and are committed to improving our manuscript accordingly.

2. Line 213, ml should be written in the same way as before.

RESPONSE:

Thank you for your attention to detail. We apologize for the inconsistency in the formatting of the unit “ml” on line 213, which should be written as “mL” and has been corrected in the revised manuscript. We have double-checked the entire manuscript to ensure accurate representation and consistent formatting of quantity units.

3. Lin133, and 200, please replace “in vivo” with “*in vivo*”.

RESPONSE:

Your meticulous review is greatly appreciated. We apologize for the inconsistency in the formatting of "in vivo" on lines 133 and 200. We have corrected this by italicizing "in vivo" and "in vitro" throughout the manuscript to ensure consistency.

4. The red fluorescence of Tp in Fig1A is not obvious.

RESPONSE:

We are grateful for your detailed observation regarding Figure 1A. We acknowledge that the red fluorescence of *Tp* may not have been clearly visible in the original manuscript. It's important to note that cells were not permeabilized during the immunofluorescence process to observe *Tp* around the cell surface and its spatial relationship with the fibronectin matrix after 6-hour infection. Typically, live *Tp* can fully or partially enter cells shortly after infection, resulting in punctate or short rod-shaped red fluorescence in the Ltp group. And dead *Tp* was washed off before fixation and staining, so the Dtp group showed no fluorescence, similar to the control group. We have taken steps to enhance the visibility of the red fluorescence in the revised figure to ensure that it is more clearly discernible.

5. Fig3C: Similarly, Less and not obvious Tp red fluorescence in this figure will affect the reliability of the experiment

RESPONSE:

Your attention to detail is greatly appreciated. In Figure 3C, we also aimed to observe *Tp* around the cell surface and its spatial relationship with the fibronectin matrix after a 6-hour infection. To achieve this, cells were not permeabilized during the immunofluorescence process. We have ensured that all such issues are corrected in the revised version of the manuscript.

Reviewer #2: (No Response)

Reviewer #3: 2. In line 204, I noticed that the decimal place in “100 μL” is not consistent with other numbers. It would be beneficial to ensure uniformity in reporting the decimal places for better clarity and accuracy.

RESPONSE:

Thank you for your meticulous review and for pointing out the inconsistency in the decimal places. We have carefully reviewed the manuscript and ensured uniformity in reporting the decimal places for better clarity and accuracy. Specifically, we have corrected the notation in line 204, changing “100 μL” with “100.0 μL”, to ensure consistency with other numerical values throughout the text.

We appreciate your attention to detail, which has helped improve the quality of our manuscript.

3. In Figure 1-6, there is an asterisk (*) or two asterisk (*) symbol, but there is no specific explanation provided in the figure caption. It would be helpful to include a clear description of what the asterisk represents in the figure.

RESPONSE:

Thank you for your valuable feedback and for highlighting the need for clarity in our figure captions. We have revised all figures to include a clear description of what the asterisk (*) and double asterisk (**) symbols represent in each figure caption.

Specifically, the asterisks indicate the levels of statistical significance: (*) denotes p < 0.05, (**) denotes p < 0.01, and (***) denotes p < 0.001. We have ensured that these explanations are now clearly provided in the figure captions.

We appreciate your attention to detail, which has helped improve the clarity and comprehensibility of our figures.

Reviewer #4: 1. Generally, the writing of figure legends and result section needs to be significantly improved. The figure legends need to include sufficient details of how the experiments are performed. The result section needs to start from describing briefly how the work is performed.

RESPONSE:

Thank you for dedicating time and effort to reviewing our manuscript. We have implemented significant improvements to address your concerns: (a) We have revised the figure legends to include detailed descriptions of the experimental procedures, ensuring clarity on how the experiments were conducted. (b) The results section has been restructured to start with a concise description of the experimental methods used, providing context for the presented findings.

These enhancements aim to facilitate readers in following the research flow and understanding the methodology underpinning our results. We appreciate your valuable feedback, which has improved the clarity and comprehensibility of our manuscript.

2. The introduction section requires to provide sufficient background the authors need to know to understand this work. The current version of introduction is not very clear. For example, it is better to discuss more details about the differences of cellular and soluble plasma FN as such differences drive the implication of the key in vivo experiment by introducing different FN to demonstrate T. pallidum penetrating mechanisms. Further, does T. pallidum bind to fibronectin? How much is known about that? In Line 92, where is that Ser39 coming from? The detailed introduction of Ser39 is needed.

RESPONSE:

Thank you for your expert opinions. We have expanded the introduction to include more detailed background information essential for understanding our work. Specifically, we have added a description of the distinctions between cellular and plasma fibronectin (FN), introduced a section discussing the interaction between *Tp* and fibronectin and its implications for the pathogen's invasive capabilities, and provided a detailed explanation of Ser39.

These improvements significantly clarify the rationale of our study, and we greatly appreciate your valuable feedback, which has been instrumental in refining our manuscript.

3. (Line 35), Typo …. test “demonstrated” that

RESPONSE:

Thank you for your careful review and for pointing out the typographical error on line 35. We have corrected the wrong typo, changing it to the correct form - "demonstrated".

We appreciate your attention to detail and your valuable feedback, which helps us improve the quality of our manuscript.

4. Throughout the manuscript, the quality of the immunofluorescent figures was very low. Additionally, no bacteria can be identified in the panel showing the staining results of T. pallidum.

RESPONSE:

Thank you for your constructive criticism regarding the quality of the immunofluorescent figures. We apologize for the low resolution of the images presented in the initial submission and have made the following improvements to address your concerns:

(a) We have replaced all low-resolution fluorescence images with high-quality images to ensure clearer and more detailed visualization.

(b) In the manuscript, we clarified that the staining of *Tp* was conducted without permeabilizing the cells after fixation. Consequently, the primary antibody for *Tp* and the corresponding fluorescent secondary antibody could only stain extracellular *Tp* or the extracellular portions of *Tp* structures. During the 6-hour infection, *Tp* could enter the cell completely or partially within a very short period of time after infection, so the red fluorescence of live *Tp* was mostly punctate or short rod-shaped in the Ltp group. In contrast, dead *Tp* was washed off before fixation and staining, and thus the Dtp group showed no fluorescence, similar to the control group.

We believe these adjustments effectively address the issues raised and enhance the overall quality of our manuscript. Thank you for your valuable feedback, which has helped us improve the presentation of our data.

5. (Line 259-260), Is there any way to quantify the clustered cFN matrix and try to do some statistical analysis?

RESPONSE:

Thank you for your suggestion regarding the quantification of the clustered cFN matrix. While we acknowledge the value of quantifying these clusters and conducting statistical analysis, we believe that such quantification is not necessary for our study. The main objective of our immunofluorescence assay is to qualitatively assess the presence and distribution of the cFN matrix. Our methodology provides sufficient detail to support our findings without the need for quantitative analysis.

The cFN matrix is inherently variable and complex, which makes accurate quantification challenging and may not yield meaningful statistical data. Immunofluorescence serves to visually demonstrate the distribution of the cFN matrix, but its qualitative nature limits absolute quantitative statistics.

Furthermore, our Western blotting (WB) results provide a more quantifiable measure of cFN levels. When combined with the visual confirmation from immunofluorescence, these findings offer a comprehensive understanding of the alterations in the cFN matrix.

We appreciate your understanding and thank you for your valuable feedback.

6. (Fig. 1B to D) Are these FN showing actually the sum of cFN and pFN so the conclusion that cFN levels are not altered is not correct? This is very important for the author to make the conclusions of certain FN making the contribution of particular phenotypes.

RESPONSE:

Thank you for your detailed review. We apologize for the misunderstanding arose from our insufficient explanation in the Introduction section. Cellular fibronectin (cFN) is typically secreted by cells (such as vascular endothelial cells and fibroblasts) and then polymerize into dimers and tetramers, which further assemble and crosslink in the extracellular matrix (ECM) to form matrix, constituting a crucial component of the ECM. In contrast, plasma fibronectin (pFN) is primarily secreted by the liver into the bloodstream, where it circulates throughout the body.

There are distinct compositional differences between cFN and pFN; cFN includes additional domains (EDA and EDB) compared to pFN. cFN can self-assemble into fibrillar matrix together with other ECM proteins, forming insoluble FN and insoluble matrix. Upon matrix disaggregation, cFN can become soluble FN. In contrast, pFN circulating in the blood does not form matrix and remains soluble.

In our study, all experiments involving Western blotting and immunofluorescence assay were conducted to investigate interactions between *Tp* and the extracellular ECM. Therefore, all measurements of total FN, insoluble FN, and soluble FN pertain to cFN specifically.

7. (Line 276, soluble and insoluble FN), The verification of no pFN is present at detectable levels in cFN would need to be provided in order for the authors to make the conclusion in the following studies that they are actually studying cFN (rather than the mixture of pFN and cFN).

RESPONSE:

Thank you for your insightful comments. As clarified earlier, the misunderstanding arose from inadequate textual descriptions. The fibronectin (FN) studied in our research, which surrounds vascular endothelial cells and forms the FN matrix, specifically refers to cellular fibronectin (cFN) rather than plasma fibronectin (pFN).

We apologize for any confusion caused and have enhanced the description of FN in the revised manuscript.

8. (Line 284), What is “its”? Is this the solubility of vimentin or FN? Additionally, in the same line, which protein is inhibited for its translocation? Vimentin or FN?

RESPONSE:

Thank you for your question regarding line 284. In this whole sentence, "its" refers to vimentin. The solubility being discussed pertains to vimentin, and it is vimentin's translocation that is being inhibited. We appreciate your attention to detail and your request for clarification. Here’s the revised text:

"According to well-established evidence, the phosphorylation of serine residues in intracellular vimentin enhances vimentin’s solubility, thereby hindering the translocation of vimentin from the perinucleus to the inner cell membrane."

9. (Fig. 2D) Please provide the arrow to specify the location of "confined vimentin distribution around the nucleus." Additionally, there seems to be one cell in the field of the cells treated with LTp. please show the field with more cells as the represented picture.

RESPONSE:

Thank you for your valuable comments regarding Fig 2D. We have revised the image to include a field with more cells treated with live *Tp* (LTp), ensuring a more representative picture. Based on this, we have added arrows to the figure to clearly indicate the location of the "confined vimentin distribution around the nucleus."

We appreciate your attention to these details and your feedback, which has contributed to enhancing the clarity and quality of our presentation.

10. (Line 317), What is AKT? This needs to be introduced in the introduction section. Is AKT AGC kinase?

RESPONSE:

Thank you for your insightful comments and for highlighting the need for clarity regarding AKT. We appreciate your attention to detail.

The AGC kinase group encompasses a subgroup of Ser/Thr protein kinases, named after three representative families: cAMP-dependent protein kinase (PKA), cGMP-dependent protein kinase (PKG), and protein kinase C (PKC). This group consists of 63 evolutionarily related serine/threonine protein kinases, that are highly conserved throughout eukaryotic evolution. Members of this group include PDK1, PKB/Akt, SGK, PKC, PRK/PKN, MSK, RSK, S6K, PKA, PKG, DMPK, MRCK, ROCK, NDR, LATS, CRIK, MAST, GRK, Sgk494, and YANK, with the Aurora and PLK families being the most closely related^[1,2]^.

Akt (including AKT1, AKT2, AKT3), also known as Protein Kinase B (PKB), plays a crucial role in various cellular processes such as glucose metabolism, cell proliferation, apoptosis, transcription, and cell migration^[3]^. We apologize for not having included a more detailed introduction of AKT1 in the initial manuscript. In response to your suggestion, we have added a comprehensive description of AKT1 in the introduction section to ensure that its significance and function are clearly communicated. Specifically, we have explained its role as an AGC kinase and its involvement in the signaling pathways relevant to our study.

References of this response:

1.	Leroux AE, Schulze JO, Biondi RM. AGC kinases, mechanisms of regulation ‎and innovative drug development. Seminars in Cancer Biology. 2018;48:1-17. doi.org/https://doi.org/10.1016/j.semcancer.2017.05.011

2.	Pearce LR, Komander D, Alessi DR. The nuts and bolts of AGC protein kinases. Nature Reviews Molecular Cell Biology. 2010;11(1):9-22. doi.org/10.1038/nrm2822

3.	Kandel ES, Hay N. The regulation and activities of the multifunctional serine/threonine kinase Akt/PKB. Experimental cell research. 1999;253(1):210-29. doi.org/10.1006/excr.1999.4690 PMID:10579924.

11. (Line 354-374), in the in vivo experiment, the cFN, laminin inoculated into rabbits were not from rabbits. How similar are the sequences from human vs. rabbit cFN or human vs. laminin? Would it be possible that those proteins trigger anti-cFN, or anti-laminin antibodies that leads to the dissemination blocking results you observed?

RESPONSE:

We appreciate your insightful comments. As shown in S2 File, we present evidence demonstrating a fairly high homology between human and rabbit FN proteins, including both the whole protein sequences and each domain. Furthermore, compared to healthy rabbits, no detectable signals of anti-FN or anti-laminin antibodies were observed in the peripheral blood of rabbits injected intradermally with FN or laminin proteins, as assessed by ELISA 26 days post-injection.
